# DNAmFitAge: biological age indicator incorporating physical fitness

**DOI:** 10.18632/aging.204538

**Published:** 2023-02-22

**Authors:** Kristen M. McGreevy, Zsolt Radak, Ferenc Torma, Matyas Jokai, Ake T. Lu, Daniel W. Belsky, Alexandra Binder, Riccardo E. Marioni, Luigi Ferrucci, Ewelina Pośpiech, Wojciech Branicki, Andrzej Ossowski, Aneta Sitek, Magdalena Spólnicka, Laura M. Raffield, Alex P. Reiner, Simon Cox, Michael Kobor, David L. Corcoran, Steve Horvath

**Affiliations:** 1 Department of Biostatistics, Fielding School of Public Health, University of California Los Angeles, Los Angeles, CA 90095, USA; 2 Research Institute of Sport Science, University of Physical Education, Budapest, Hungary; 3 San Diego Institute of Science, Altos Labs, San Diego, CA 92121, USA; 4 Department of Epidemiology and Butler Columbia Aging Center, Columbia University Mailman School of Public Health, New York, NY 10032, USA; 5 Department of Cancer Epidemiology, University of Hawaii, Honolulu, HI 96813, USA; 6 Centre for Genomic and Experimental Medicine, Institute of Genetics and Cancer, University of Edinburgh, Edinburgh EH4 2XU, UK; 7 Longitudinal Studies Section, Translational Gerontology Branch, National Institute on Aging, National Institutes of Health, Baltimore, MD 21224, USA; 8 Małopolska Centre of Biotechnology, Jagiellonian University, Kraków, Poland; 9 Department of Forensic Genetics, Pomeranian Medical University in Szczecin, Szczecin, Poland; 10 Institute of Zoology and Biomedical Research, Faculty of Biology, Jagiellonian University, Kraków, Poland; 11 Department of Anthropology, Faculty of Biology and Environmental Protection, University of Łódź, Łódź, Poland; 12 Central Forensic Laboratory of the Police, Warsaw, Poland; 13 Department of Genetics, University of North Carolina at Chapel Hill, Chapel Hill, NC 27599, USA; 14 Department of Epidemiology, University of Washington, Seattle, WA 98195, USA; 15 Lothian Birth Cohorts, Department of Psychology, University of Edinburgh, Edinburgh EH8 9JZ, UK; 16 Department of Medical Genetics, University of British Columbia, Vancouver, Canada; 17 Lineberger Comprehensive Cancer Center, University of North Carolina at Chapel Hill, Chapel Hill, NC 27599, USA

**Keywords:** epigenetics, aging, physical fitness, biological age, DNA methylation

## Abstract

Physical fitness is a well-known correlate of health and the aging process and DNA methylation (DNAm) data can capture aging via epigenetic clocks. However, current epigenetic clocks did not yet use measures of mobility, strength, lung, or endurance fitness in their construction. We develop blood-based DNAm biomarkers for fitness parameters gait speed (walking speed), maximum handgrip strength, forced expiratory volume in one second (FEV1), and maximal oxygen uptake (VO2max) which have modest correlation with fitness parameters in five large-scale validation datasets (average r between 0.16–0.48). We then use these DNAm fitness parameter biomarkers with DNAmGrimAge, a DNAm mortality risk estimate, to construct DNAmFitAge, a new biological age indicator that incorporates physical fitness. DNAmFitAge is associated with low-intermediate physical activity levels across validation datasets (*p* = 6.4E-13), and younger/fitter DNAmFitAge corresponds to stronger DNAm fitness parameters in both males and females. DNAmFitAge is lower (*p* = 0.046) and DNAmVO2max is higher (*p* = 0.023) in male body builders compared to controls. Physically fit people have a younger DNAmFitAge and experience better age-related outcomes: lower mortality risk (*p* = 7.2E-51), coronary heart disease risk (*p* = 2.6E-8), and increased disease-free status (*p* = 1.1E-7). These new DNAm biomarkers provide researchers a new method to incorporate physical fitness into epigenetic clocks.

## INTRODUCTION

Physical fitness declines with aging and is well known to correlate to health [1]. This decline is evident in reduced function in specific organs, like lungs [2], and in performance tests of strength [3] or aerobic capacity [4]. The rate of this decline varies between individuals [5, 6], and those who preserve physical fitness as they age are at lower risk for a range of diseases and tend to live longer lives [6–8]. At the molecular level, changes in fitness and related indices of functional capacity correlate with changes in molecular signs of decline thought to reflect underlying biological processes of aging [9]. Measures of fitness may therefore provide a new window into biological aging [10]. However, direct measurement of fitness parameters can be challenging, requiring in-person data collection by trained personnel with specialized equipment [11]. Furthermore, fitness measurements are not possible for studies with remote data collection or those conducted with stored biospecimens. To enable such studies to quantify fitness, we developed blood based DNAm biomarkers of fitness parameters spanning mobility, strength, lung function, and cardiovascular fitness and use these to construct a novel indicator of fitness-based biological age, DNAmFitAge.

Three lines of evidence support a focus on DNAm to develop biomarkers of fitness and aging-related changes in fitness. First, aging is reflected in DNAm changes; hundreds of thousands of CpG sites across the genome change methylation states as organisms grow older, enabling construction of high-precision algorithms to predict age [12, 13]. These are collectively known as epigenetic clocks, and a large body of literature demonstrates these clocks are associated with human mortality risk [14, 15], various age-related conditions [15–17], and are reflective of one’s biological age [14, 17]. Second, prediction of aging-related morbidity, disability, and mortality by DNAm biomarkers is enhanced by the incorporation of physiological data, like smoking pack years and white blood cell counts, into prediction algorithms [14, 15, 18]. This suggests utility in including physical fitness in DNAm biomarkers, however, current DNAm biomarkers do not use fitness parameters in their construction. Third, there is emerging evidence that epigenetic clocks are sensitive to lifestyle factors [19], individual differences in fitness parameters are reflected in DNAm data [20, 21], and blood DNAm differs between athletes and controls [22]. Therefore, a growing body of evidence suggests blood DNAm carries information related to physical fitness, but it was unknown if fitness parameters could be estimated using blood DNAm levels.

Here, we develop blood DNAm biomarkers of four fitness parameters: gait speed (walking speed), maximum handgrip strength, forced expiratory volume in 1 second (FEV1; an index of lung function), and maximal oxygen uptake (VO2max; a measure of cardiorespiratory fitness). These parameters were chosen because handgrip strength and VO2max provide insight into the two main categories of fitness: strength and endurance [23], and gait speed and FEV1 provide insight into fitness-related organ function: mobility and lung function [8, 24]. Furthermore, each parameter is commonly measured and known to be associated with aging, mortality, and disease [8, 24–26]. We then use these DNAm fitness biomarkers to develop the novel DNAm fitness-related biological age indicator, DNAmFitAge, which quantifies the relationship between physical fitness and biological aging processes. This novel measure incorporates mortality risk with strength, mobility, and cardiovascular fitness using blood DNAm biomarkers. Our newly constructed DNAm biomarkers and DNAmFitAge provide researchers and physicians a new method to incorporate physical fitness into epigenetic clocks and emphasizes the effect lifestyle has on the aging methylome.

## RESULTS

### DNAm fitness parameter biomarker models

**Figure 1:**
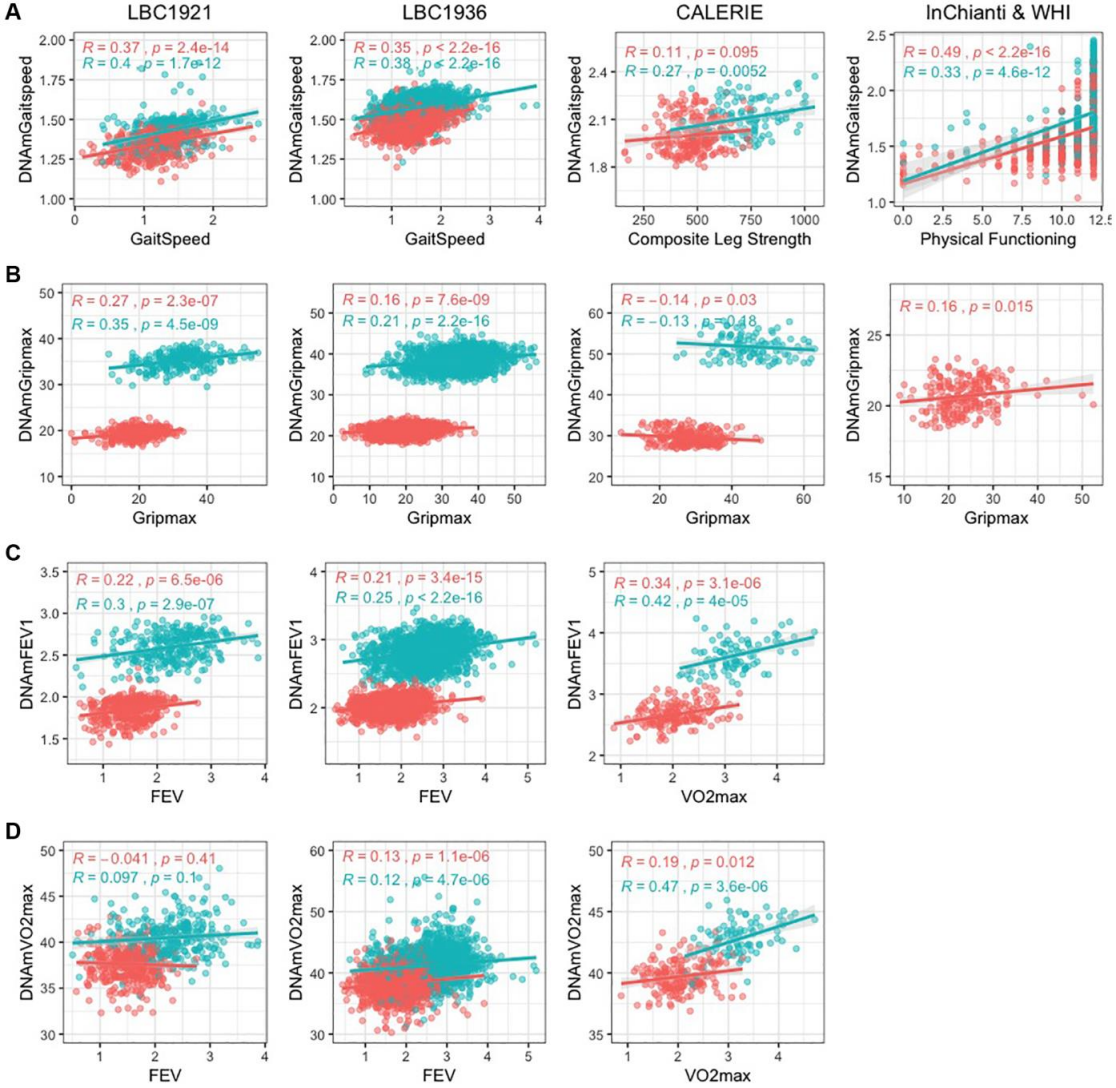
**Scatterplots of DNAm fitness biomarker models versus true values in test datasets.** Pink indicates females, and blue indicates males. When original variables were unavailable, best alternative variables are plotted against the DNAm fitness estimates. Each panel corresponds to the performance of one DNAm-based model built with chronological age across test datasets displayed with Pearson correlation and *p*-values. (**A**) DNAmGaitspeed with performance in InChianti dataset displayed, (**B**) DNAmGripmax with performance in WHI dataset, (**C**) DNAmFEV1, (**D**) DNAmVO2max. (**A**–**C**) (DNAmGaitspeed, DNAmGripmax, and DNAmFEV1) were built in each sex separately while (**D**) (DNAmVO2max) was built in both sexes jointly.

The DNAm fitness parameter biomarkers built with blood DNA methylation had modest correlation with direct fitness parameters. Average correlations across validation datasets ranged from 0.16–0.48 (Figure 1, Table 1). DNAmGripmax in males and females had moderate correlations in validation datasets but do not perform well in CALERIE. We hypothesize this may be due in part from the stringent enrollment criteria: free of chronic disease, non-obese, and relatively young, which yields less variation in fitness measures. Correlation of DNAmVO2max to FEV in LBC1921 and LBC1936 was weak within each sex, likely caused by the small age range in each cohort; however varying correlations between FEV and VO2max have also been described in literature [27–29]. Reported correlations between VO2max and FEV vary from 0 to 0.5, likely because VO2max is a measure of cardiovascular health whereas FEV is a measure of lung volume. Correlation of DNAmVO2max to VO2max in CALERIE, the one validation dataset with the same direct fitness parameter, has good correlation overall and within sex (overall R = 0.55, female R = 0.19, male R = 0.47).

**Table 1:** DNAm fitness parameter biomarker Pearson correlation.

**DNAm biomarker**	**CpG**	**Age in model**	**Sex**	**FHS + BLSA**	**Budapest**	**LBC 1921**	**LBC 1936**	**CALERIE**	**InChianti**	**WHI**	**Average test R**
Gait speed	42	Y	Females	*0.61*	0.61	0.37	0.34	0.11^*^	0.49^+^	0.15^+^	0.34
	26	Y	Males	*0.43*	0.59	0.40	0.38	0.27^*^	0.33^+^		0.39
	53	N	Females	*0.56*	0.56'	0.17	0.17	0.095^*^	0.43^+^	0.12^+^	0.26
	59	N	Males	*0.60*	0.53'	0.23	0.21	0.26^*^	0.34^+^		0.31
Gripmax	52	Y	Females	*0.66*	0.54	0.27	0.16	−0.14		0.16	0.20
	52	Y	Males	*0.68*	0.50	0.35	0.19	−0.089			0.24
	91	N	Females	*0.66*	0.52	0.22	0.10	−0.16		0.12	0.16
	93	N	Males	*0.66*	0.43	0.21	0.14	−0.078			0.18
FEV1	77	Y	Females	*0.59*	0.50^v^	0.21^^^	0.20^^^	0.34			0.31
	73	Y	Males	*0.63*	0.30^v^	0.30^^^	0.25^^^	0.42			0.32
VO2max	40	Y	Both	0.52^$^	*0.70*	0.43^^^	0.40^^^	0.55			0.48

Superscripts indicate correlation is with the closest fitness parameter available: 'Jumpmax, ^*^Composite Leg Strength, ^+^Physical Functioning, ^^^FEV, ^$^FEV1, ^v^VO2max. Gray italics denote training dataset.

The DNAm biomarkers improve estimation of fitness parameters beyond what is explained through age and sex in many validation datasets (Supplementary Table 1). Table 1 shows between 26 and 93 CpG loci were selected through LASSO to estimate each fitness parameter. Without age as a covariate in the DNAm biomarker estimates, more CpG loci were needed to achieve similar precision- between 53 and 93. DNAmVO2max model includes several CpG loci on the X chromosome, likely capturing sex effects. Interestingly, DNAmGaitspeed and DNAmGripmax built without chronological age have lower correlation with true fitness parameters compared to the models built with chronological age, however these biomarkers explain more additional variation in fitness parameters compared to the age-included versions. This suggests the DNAm biomarkers capture different information than age and sex for understanding fitness parameters. R code to calculate DNAm fitness biomarkers is available in our GitHub repository at https://github.com/kristenmcgreevy/DNAmFitAge.

### DNAm fitness biomarkers in age-related conditions

All DNAm fitness biomarkers are individually predictive of mortality and disease-free status, and some are predictive of type 2 diabetes status and number of comorbidities in the validation datasets. After controlling for age and sex, higher (or more fit) values of DNAmGaitspeed without age (*p* = 1.1E-10), DNAmGripmax without age (*p* = 2.6E-9), DNAmFEV1 (*p* = 2.2E-20), and DNAmVO2max (*p* = 0.003) are associated with decreasing mortality risk (Supplementary Figure 1). For example, on average, every 1 kg stronger DNAmGripmax is has an associated 5% decrease in mortality risk compared to a person of the same age and sex (hazard ratio = 0.95, confidence interval = [0.93, 0.96]). DNAmGaitspeed and DNAmFEV1 are both predictive of type 2 diabetes status (*p* = 0.0013, *p* = 0.0032) and number of comorbidities (*p* = 0.0004, *p* = 4E-12). Stronger values of any DNAm fitness biomarkers are associated with disease-free status. Relationship of each DNAm fitness biomarker with time-to-death, type 2 diabetes, number of comorbidities, and disease-free status after adjusting for age and sex are displayed in Supplementary Figure 1. Relationship of DNAm biomarkers to physical activity are explored alongside DNAmFitAge below.

### DNAmFitAge

**Figure 2:**
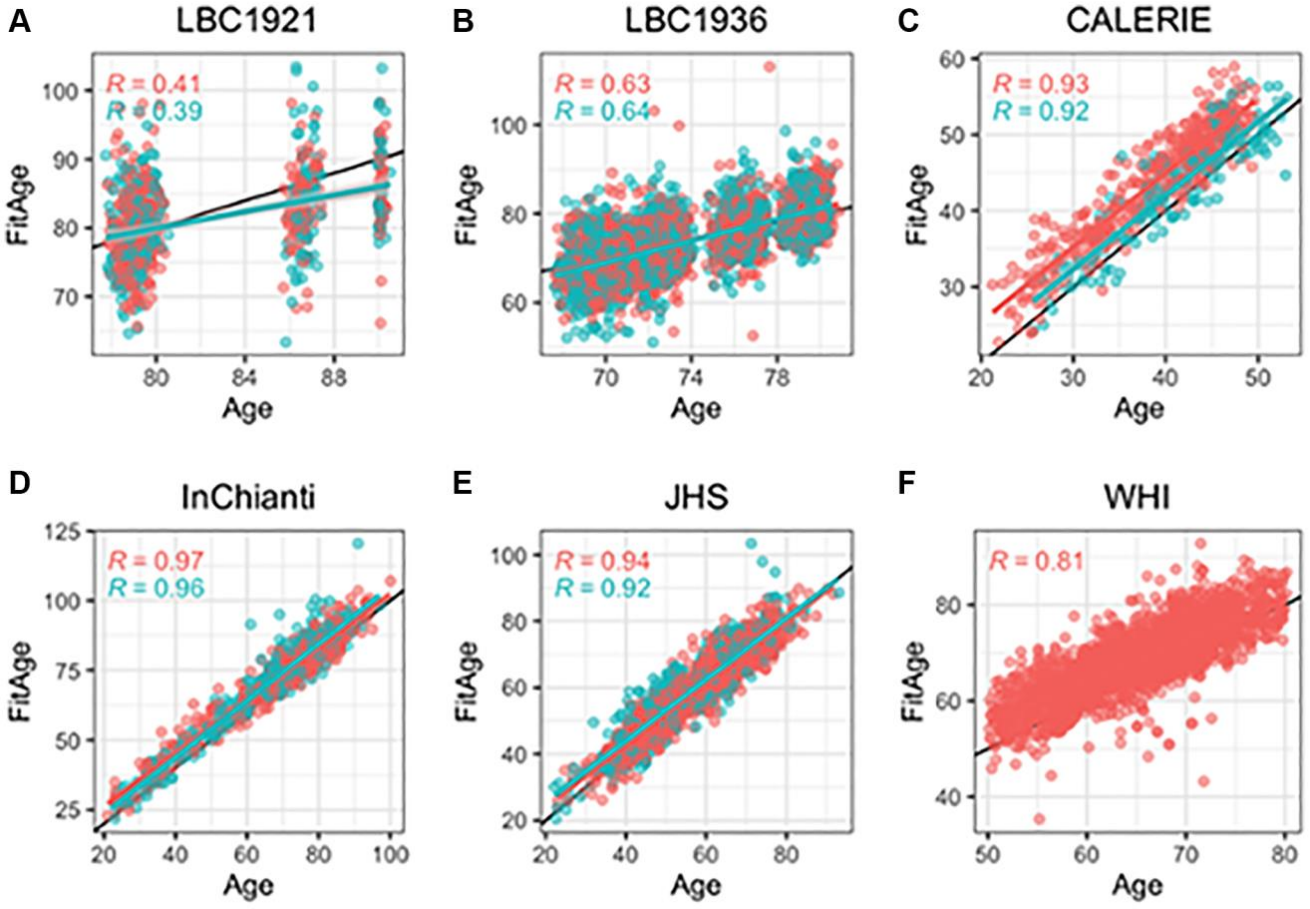
**Scatterplots of DNAmFitAge versus age separated by sex.** Pink indicates females, and blue indicates males. (**A**–**F**) Each panel corresponds to the performance of DNAmFitAge in one validation dataset displayed with Pearson correlation to chronological age and corresponding *p*-values. DNAmFitAge models applied to the same sex it was built in (i.e., DNAmFitAge built for females tested in females and DNAmFitAge built for males tested in males). DNAmFitAge is centered on chronological age with high correlation across all test sets.

DNAmFitAge provides an estimate of biological age, and FitAgeAcceleration is a measure of epigenetic age acceleration. DNAmFitAge had strong correlation to chronological age in validation datasets. The average Pearson r between DNAmFitAge and chronological age across validation datasets was 0.77 (Figure 2), and the lower correlation in LBC1921 (r = 0.38) and LBC1936 (r = 0.68) can be attributed to the small age range they cover. LBC1921 ages ranged from 77 to 90 and LBC1936 ages ranged from 67 to 80. The average r excluding LBC cohorts was 0.92. The DNAm fitness biomarkers contribute 319 unique CpG loci to construct DNAmFitAge, and the contribution among DNAm fitness biomarkers were very similar in males (13.9–17.9%) with slightly more variation in females (10.4–22.4%). DNAmGripmax, DNAmVO2max, and DNAmGaitspeed contributed around 50% to estimating DNAmFitAge in each sex, and DNAmGrimAge contributed the remaining 50% (Table 2A). In addition, each validation dataset had low median absolute deviation (median of the absolute difference from chronological age to biological age) ranging from 2.3 to 4.9 years (Supplementary Table 2). Reproducibility across a wide span of ages (21 in CALERIE to 100 in InChianti) demonstrate DNAmFitAge’s calibration across a wide adult age range.

**Table 2A:** DNAmFitAge model weights.

**Variable**	**Female weights**	**Male weights**
DNAmGripmax	0.174	0.179
DNAmGaitSpeed	0.228	0.159
DNAmVO2max	0.104	0.139
DNAmGrimAge	0.493	0.523

Applying each DNAmFitAge model to the opposite sex shows strong correlation with age but with substantial over and underestimation of age in females and males, respectively (Supplementary Table 2, panels B–H Supplementary Figure 2). Over and under estimation are explained by universal differences in male and female fitness parameters. Females tend to have lower fitness parameters compared to males. Hence males were predicted to be younger than they are using the female DNAmFitAge model because larger values of DNAmGaitSpeed, DNAmGripmax, or DNAmVO2max indicates stronger (or more physically fit) females.

Reference DNAm fitness biomarker values corresponding to fit DNAmFitAge compared to unfit DNAmFitAge within age and sex categories are provided in Table 2B. “Fit” corresponds to biological age being 5 years younger than expected (FitAgeAcceleration ≤ −5), and “unfit” corresponds to biological age being 5 years older than expected. Average differences (Fit – Unfit) across all age and sex categories are 0.2 m/s faster DNAmGaitSpeed, 5.1 kg stronger DNAmGripmax, and 2.0 mL/kg/min better DNAmVO2max. Overall, higher or more physically fit values of DNAmGaitspeed, DNAmGripmax, or DNAmVO2max correspond to younger estimated biological ages in males and females.

**Table 2B:** Reference DNAm fitness parameter values for fit (FitAge acceleration <= −5 yrs) and unfit (FitAge Acceleration >= +5 yrs) individuals.

**Age**	**Females**							
	**DNAmGaitspeed**		**DNAmGripmax**		**DNAmVO2max**		**DNAmGrimAge**	
**Fit**	**Unfit**	**Fit**	**Unfit**	**Fit**	**Unfit**	**Fit**	**Unfit**
<40	2.1	2.0	34.6	30.5	42.8	40.1	37.1	40.9
40–59	1.9	1.7	31.3	26.9	39.2	37.9	49.2	60.5
60–79	1.7	1.5	28.8	22.4	37.6	36.1	63.2	72.4
80+	1.6	1.3	23.9	19.1	37.0	35.4	74.7	81.8
**Age**	**Males**							
	**DNAmGaitspeed**		**DNAmGripmax**		**DNAmVO2max**		**DNAmGrimAge**	
**Fit**	**Unfit**	**Fit**	**Unfit**	**Fit**	**Unfit**	**Fit**	**Unfit**
<40	2.1	1.8	49.3	43.8	45.1	44.9	34.8	52.9
40–59	1.9	1.7	46.6	42.5	43.9	42.3	47.5	60.1
60–79	1.7	1.5	41.3	36.8	43.1	39.5	68.0	77.9
80+	1.6	1.3	39.3	32.0	41.3	37.7	78.0	86.7

### FitAgeAcceleration in age-related conditions

**Figure 3:**
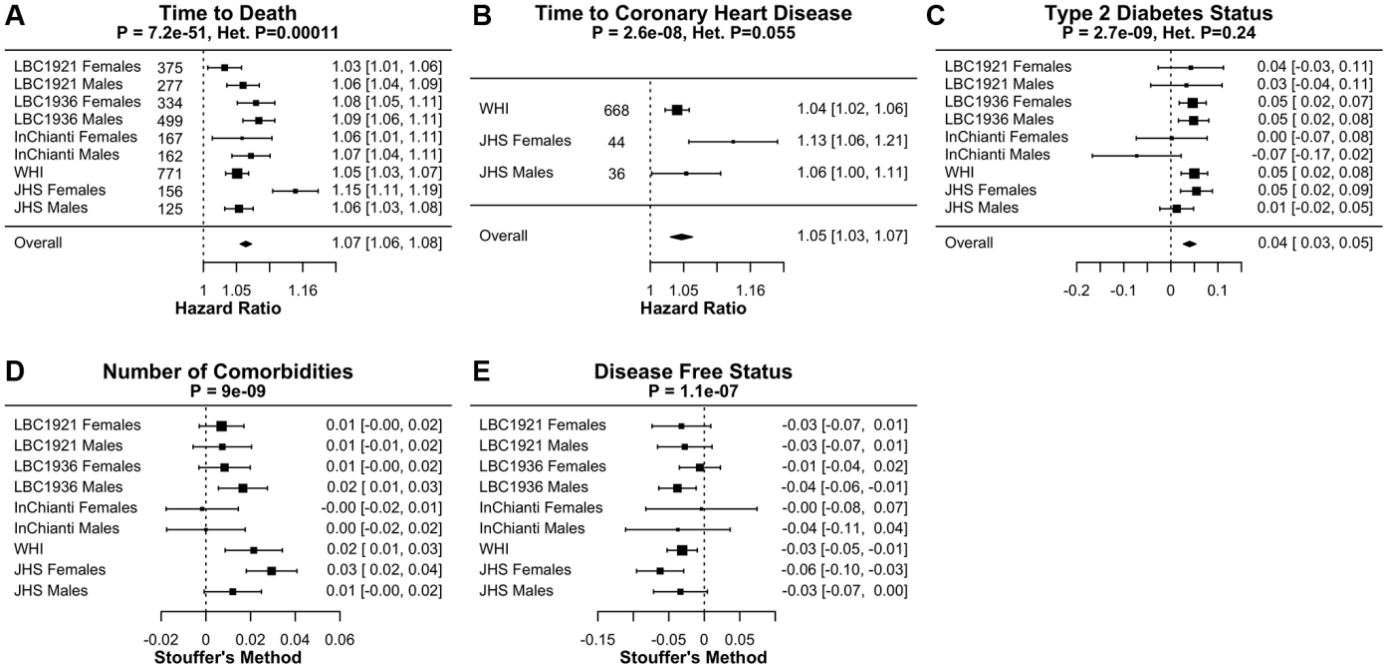
**Meta-analysis forest plots for FitAgeAcceleration to age-related conditions adjusted for age and sex.** Each panel reports a meta analysis forest plot for combining hazard ratios or regression coefficients across dataset cohorts. (**A**) Time-to-death with number of events, (**B**) time-to-coronary heart disease with number of events, (**C**) type 2 diabetes, (**D**) comorbidity count, and (**E**) disease free status. Meta-analysis *p*-values are displayed in the header of each panel, and test of heterogeneity Cochran *Q* test *p*-value (Het. P) are displayed for fixed effect models. Fixed effects models were used for (**A**–**C**) and Stouffer’s method was used for (**D**, **E**).

We find that the age-adjusted version of FitAge, FitAgeAcceleration, is a significant predictor of mortality risk (all cause mortality), coronary heart disease, and other age-related conditions. Cox Proportional Hazard models demonstrated FitAgeAcceleration is a strong predictor for time-to-death (*p* = 7.2E-51) and time-to-coronary heart disease (*p* = 2.6E-8). FitAgeAcceleration had an overall hazard ratio of 1.07 (1.06, 1.08) (Figure 3). Thus, a FitAgeAcceleration value of 10 years was associated with almost doubling the mortality risk compared to the average person of the same age and sex (1.07^10^ = 1.97 risk). Similarly, increase in FitAgeAcceleration corresponds to more comorbidities (*p* = 9.0E-9), hypertension (*p* = 8.7E-5), and earlier age at menopause (*p* = 6.6E-9) (Figure 3, Supplementary Table 3). A lower FitAgeAcceleration was associated with disease free status (*p* = 1.1E-7) and lower cholesterol (*p* = 0.0005) (Supplementary Table 3).

Each of these associations were in the expected direction, as someone who had a low FitAgeAcceleration had a biological age estimate that was younger than their chronological age. Hence, people whose DNAm predicted them to be more ‘physically fit’ than their chronological age would suggest had better age-related outcomes. These relationships demonstrate epigenetic age acceleration can be well explained through DNAm fitness parameter biomarkers, and that FitAgeAcceleration provides a practical tool for relating fitness to the aging process.

FitAgeAcceleration is additionally informative for mortality risk beyond the information captured with AgeAccelGrim in JHS females and in InChianti males and females when comparing LRT *p*-values (Supplementary Table 4). FitAge Acceleration is almost always additionally informative for time-to-death compared to other epigenetic clocks, but FitAgeAcceleration is only sometimes informative beyond the epigenetic clocks for explaining number of comorbidities. Overall, our results indicate FitAgeAcceleration is informative for mortality risk and may act as a supplement (not replacement) to AgeAccelGrim.

### DNAmFitAge relationship to physical activity

**Figure 4:**
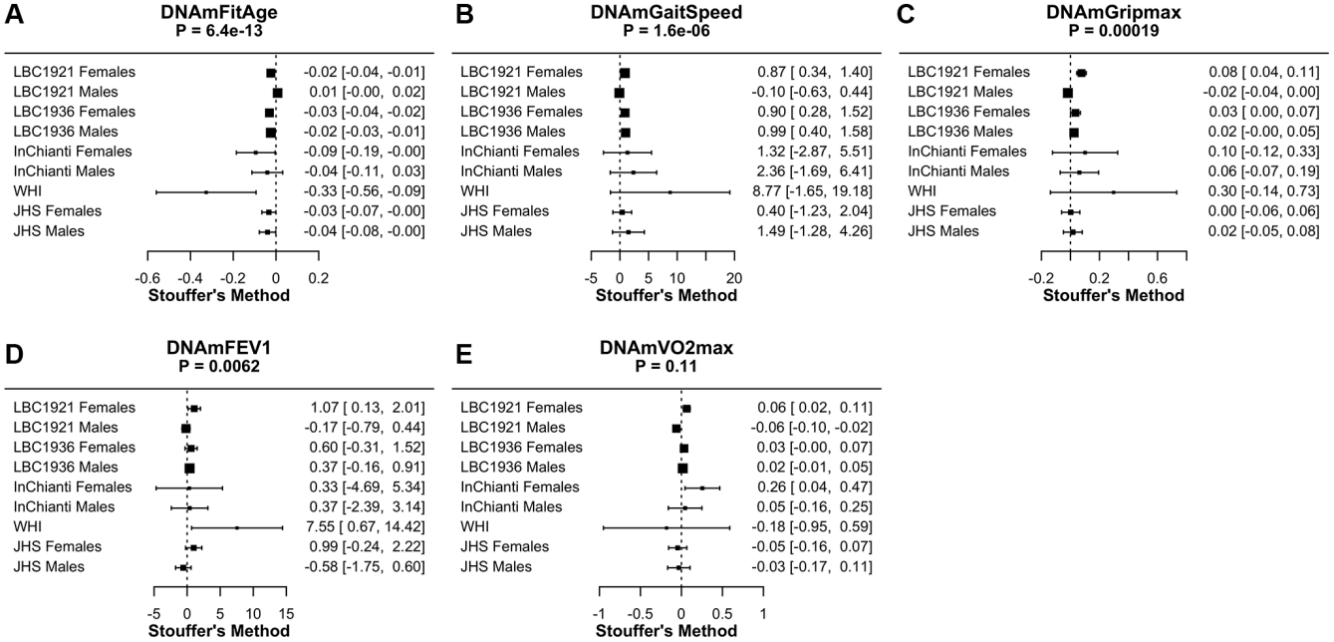
**Meta-analysis forest plots for DNAmFitAge and DNAm fitness parameters relationship to physical activity or physical functioning in people with low to intermediate physical activity.** Each panel reports the Stouffer’s meta-analysis *p*-value for combining coefficients across dataset cohorts after adjusting for chronological age. (**A**) DNAmFitAge, (**B**) DNAmGaitspeed, (**C**) DNAmGripmax, (**D**) DNAmFEV1, and (**E**) DNAmVO2max. DNAmFitAge, DNAmGaitSpeed, DNAmGripmax, and DNAmFEV1 are predictive of physical activity in low to intermediate physically active individuals.

FitAgeAcceleration, DNAmGaitspeed, DNAmGripmax, and DNAmFEV1 have associations in the expected direction with physical activity in low to intermediate physically active individuals. Coefficients indicate the effect on physical activity for a one unit increase in each DNAm fitness biomarker after adjusting for chronological age within each sex (Table 3, Figure 4). The relationship to DNAmFitAge is as expected; someone with a higher FitAgeAcceleration has an estimated biological age that is older than expected, which corresponds to lower physical activity or physical functioning (Table 2B). Similarly, men and women with a faster DNAmGaitspeed, stronger DNAmGripmax, and larger DNAmFEV1 are more physically active when holding age constant. In conclusion, men and women who were more active showed correspondingly ‘fitter’ values of FitAgeAcceleration and the DNAm fitness biomarkers. Research suggests any exercise compared to none is beneficial to health [30], and we hope DNAmFitAge may serve as a tool to motivate starting an exercise regimen at any level.

**Table 3:** Association of DNAm biomarkers to physical activity and physical functioning in people with low to intermediate activity levels.

**Outcome**		**Females**					**Males**				**Meta analysis *p*-value**
		**LBC 1921**	**LBC 1936**	**InChianti**	**JHS**	**WHI**	**LBC 1921**	**LBC 1936**	**InChianti**	**JHS**	
DNAmFitAge	coefficient	−0.024	−0.031	−0.095	−0.033	−0.237	0.008	−0.024	−0.041	−0.040	6.37E-13
	*p*-value	2.3E-04	3.7E-06	0.042	0.046	0.014	0.199	2.0E-05	0.272	0.044	
DNAmGaitSpeed w/ Age	coefficient	−0.51	2.82	8.76	3.07	26.67	−3.31	1.99	4.97	1.78	1.82E-03
	*p*-value	0.567	0.002	0.084	0.165	0.025	2.4E-04	0.022	0.429	0.672	
DNAmGaitSpeed w/o Age	coefficient	0.87	0.90	1.32	0.40	8.77	−0.10	0.99	2.36	1.49	1.60E-06
	*p*-value	0.001	0.004	0.536	0.627	0.099	0.725	0.001	0.255	0.293	
DNAmGripmax w/Age	coefficient	0.10	−0.06	0.14	-0.03	0.24	0.00	0.03	0.17	0.02	0.029
	*p*-value	0.036	0.201	0.635	0.821	0.035	0.943	0.256	0.291	0.801	
DNAmGripmax w/o Age	coefficient	0.076	0.035	0.10	0.002	0.30	−0.02	0.02	0.06	0.02	1.85E-04
	*p*-value	4.3E-06	0.043	0.379	0.953	0.181	0.125	0.058	0.364	0.617	
DNAmFEV1	coefficient	1.07	0.60	0.33	0.99	4.98	−0.17	0.37	0.37	−0.58	0.0062
	*p*-value	0.026	0.197	0.898	0.114	0.005	0.585	0.173	0.791	0.337	
DNAmVO2max	coefficient	0.06	0.03	0.26	−0.05	−0.47	−0.06	0.02	0.05	−0.03	0.113
	*p*-value	0.003	0.090	0.019	0.423	0.215	0.002	0.281	0.667	0.654	
DNAmGrimAge	coefficient	−0.01	−0.03	−0.08	−0.05	−0.30	-0.01	−0.03	-0.01	−0.05	1.25E-12
	*p*-value	0.524	5.7E-06	0.157	0.002	0.027	0.390	2.3E-05	0.794	0.007	
DNAmPhenoAge	coefficient	0.00	−0.01	−0.12	-0.02	−0.07	7.1E-05	−0.01	-0.01	−0.01	1.26E-06
	*p*-value	0.568	0.012	4.2E-04	0.063	0.354	0.989	0.004	0.764	0.425	
DNAmPAI1	coefficient	−3.4E-05	−4.4E-05	−7.5E-05	−1.1E-04	−3.4E-04	−1.8E-06	−2.8E-05	7.5E-05	−6.2E-05	6.36E-10
	*p*-value	0.021	0.002	0.358	2.5E-08	0.076	0.908	0.032	0.382	0.009	
DNAmGDF15	coefficient	−8.5E-05	−1.3E-03	-2.9E-03	−8.5E-04	−1.0E-02	−1.2E-03	−5.9E-04	−3.5E-03	−6.7E-04	6.16E-08
	*p*-value	0.802	0.0005	0.082	0.147	0.047	5.4E-04	0.054	0.117	0.373	

Additionally, DNAmFitAge (Stouffer *p*-value = 6.4E-13) marginally outperforms current DNAm biomarkers when comparing meta-analysis *p*-values; improvement of DNAmFitAge compared to DNAmGrimAge (*p*-value = 1.2E-12) is marginal, however the improvement compared to DNAmPhenoAge (*p*-value = 1.3E-6) and DNAmGDF-15 (*p*-value = 6.2E-8) is more pronounced. In addition, DNAmFitAge, which provides an indicator of biological age, may provide a more interpretable aging biomarker compared to DNAmGrimAge, which provides a measurement of lifespan. These comparisons demonstrate DNAmFitAge can capture the relationship to physical activity and can provide an improvement to the arsenal of current DNAm biomarkers.

### DNAmFitAge relationship in body builders

**Figure 5:**
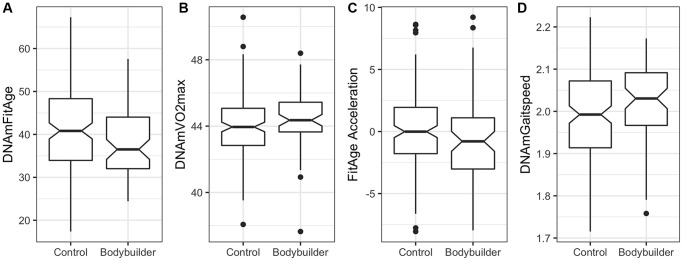
**Boxplots showing spread of DNAm biomarkers between male controls (*n* = 149) and male body builders (*n* = 66) in the Polish study.** (**A**) DNAmFitAge is younger on average in the male body builders, (**B**) DNAmVO2max is fitter on average in the male body builders, (**C**) FitAge Acceleration and (**D**) DNAmGaitspeed are suggestively improved in body builders but not significantly different at 0.05.

Male body builders are estimated to be biologically younger and more physically fit compared to male controls of the same age. On average, DNAmFitAge is 2.74 years younger in male body builders compared to controls (*p* = 0.041), and DNAmVO2max is 0.4 mL/kg/sec better in male body builders (*p* = 0.023) (Table 4). FitAge Acceleration (*p* = 0.080), DNAmGaitspeed (*p* = 0.055), and DNAmGripmax (*p* = 0.075) are suggestive of having improvement in male body builders, however they were not significant at the 0.05 level. Boxplots displaying the spread of DNAmFitAge, DNAmVO2max, FitAge Acceleration, and DNAmGaitspeed between body builders and controls are presented in Figure 5. Male body builders have 5.4 more years of regular training (*p* = 2.6E-6) and 1.1 more training sessions per week (*p* = 9.4E-7) compared to male controls on average, and the DNAmFitAge and DNAmVO2max results correspond to male body builders being estimated as more physically fit, as expected. The study was underpowered for females with only 30 female body builders, however we did examine the relationship of the DNAm fitness biomarkers in females and expectedly (due to the small sample size) did not find a significant difference between female body builders and controls. Our promising results in male body builders show a physically fit lifestyle corresponds to biological aging benefits that can be captured with our new DNAm fitness biomarkers and DNAmFitAge.

**Table 4:** Comparison between male controls and body builders in Polish study.

	**Mean control**	**Mean body builder**	**Control - body builder**	**Kruskal wallis *p*-value**
	**(*n* = 149)**	**(*n* = 66)**		
Intensity trainings per week	3	4.1	−1.1	9.43E-07
Years regular training	6.6	12	−5.4	2.61E-06
DNAmFitAge	41.1	38.4	2.74	0.041
FitAgeAcceleration	0.15	−0.56	0.72	0.08
DNAmGaitspeed	1.99	2.02	−0.03	0.055
DNAmGripmax	46.5	47.2	−0.69	0.075
DNAmVO2max	44	44.4	−0.4	0.023
DNAmFEV1	3.82	3.87	−0.05	0.199
DNAmGrimAge	44.1	41.8	2.24	0.063
DNAmPhenoAge	26.7	24.7	2.01	0.181
DNAmPAI1	19033	18238	795	0.009
DNAmGDF15	701.8	680.4	21.4	0.447

Dietary supplement use cannot explain improvement in DNAmFitAge, but multivitamin dietary supplements are associated with improvement in DNAmVO2max after controlling for athlete status and age in males. Males from the Polish Study who take multivitamins have a 0.68 mL/kg/sec fitter DNAmVO2max on average after adjusting for athlete status and age (*p* = 0.041, Supplementary Table 5). Multivitamins, energy, vitamin D, and Omega-3 all are disproportionately taken by the male body builders (Supplementary Table 6), however, supplement use is not sufficient to explain younger DNAmFitAge regardless of athlete status (Supplementary Table 5). These insignificant results may point to other components of athleticism that contribute to younger estimated biological ages, such as increased physical activity and decreased body fat. We note that supplement and athlete coefficients for multivitamins, proteins, and Omega-3 are statistically insignificant, but their relationships are in the expected direction for DNAmFitAge and DNAmVO2max. Our research does not establish the causative relationship of body building or supplement use on biological aging, but it does establish there are observable epigenetic benefits associated with being a male body builder.

### Functional CpG annotation

The 627 CpG genomic locations used to construct the DNAm biomarker estimates were enriched in 5 gene sets, 11 cellular processes, and 7 molecular processes mostly related to inflammation at FDR Q-value < 0.05. Top enrichment results from GREAT analysis passing the Bonferroni *p*-value threshold of 0.05 are presented in Table 5A and complete GREAT results are presented in Supplementary Table 7. The top genes enriched include zinc ribbon domain containing 1 (ZNRD1; Bonferroni *p* = 0.005) and histocompatibility antigen (HLA-G; *p* = 0.02). Cellular processes relate to major histocompatibility complex (MHC) proteins (*p* = 3.1E-7) and molecular processes relate to peptide antigen binding (*p* = 0.032) and tapasin binding (*p* = 0.047). Tapasin is a MHC class I antigen-processing molecule present in the lumen of the endoplasmic reticulum [31]. The relationship to inflammation-based genes and processes like HLA, MHC, and tapasin support hypotheses relating physical fitness and systemic inflammation [32]. In addition, previous research found inflammation response and endoplasmic reticulum stress were down-regulated in people following a 12-week endurance exercise regime compared to the non-exercising control group [33]. Both biological findings are intriguing and may provide direction for studying modifiable methylation from fitness parameters.

**Table 5A:** Top GREAT CpG annotation results.

	**Observed regions**	**Fold enrichment**	**Binomial *p*-value**	**Bonferroni *p*-value**
Genes				
ZNRD1	4	77.9	2.75E-07	0.0051
HLA-G	4	55.0	1.09E-06	0.020
Cellular				
MHC protein complex	9	25.1	1.86E-10	3.11E-07
Integral component of endoplasmic reticulum membrane	21	3.7	4.49E-07	0.00075
Intrinsic component of endoplasmic reticulum membrane	21	3.6	6.39E-07	0.0011
MHC class II protein complex	5	26.9	1.56E-06	0.0026
Integral component of lumenal side of Endoplasmic reticulum membrane	7	12.7	1.81E-06	0.0030
Molecular				
Peptide antigen binding	6	13.3	7.71E-06	0.032
Tapasin binding	2	421.0	1.12E-05	0.047

Next, we examined the chromatin states of the genomic regions across the 627 CpG sites used for DNAm fitness biomarker construction and found CpG loci are significantly depleted in heavily acetylated promoters and transcription start sites (TSS) and enriched in regions with polycomb repressive complex 2 (PRC2) binding. The odds ratios (OR) are significantly less than one in the chromatin state PromF4 (heavily acetylated promoters, OR = 0.45, hypergeometric *p* = 6.5E-6) and TSS1 (acetylated TSS, OR = 0.37, *p* = 6.8E-6) (Table 5B). BivProm1 (OR = 1.50, *p* = 0.009), BivProm2 (OR = 1.76, *p* = 0.0006), and ReprPC1 (OR = 1.87, *p* = 0.007) regions are enriched in our DNAm fitness biomarkers and are known PRC2 binding sites [34]. BivProm1 and BivProm2 are weak bivalent promoters and ReprPC1 is a polycomb repressed region. Bivalent chromatin domains control expression of *HOX* and other developmental genes in all vertebrates. PRC2 is one of the main Polycomb repressive complexes (PRC) that act as negative epigenetic regulators of transcription; it helps to initiate gene silencing via H3K27 methylation [35]. These results coincide with the increasing observation that the process of development is connected to epigenetic aging and that PRC2 targets are enriched in the age-dependent methylome in human and mammals [12, 36].

**Table 5B:** Chromatin state enrichment.

**State**	**Description**	**Number of CpG loci**	**Odds ratio**	**Hypergeometric *p*-value**
PromF4	Promoter; heavily acetylated - flanking tss downstream bias	25	0.45	6.5E-06
TSS1	TSS more acetylated and active	15	0.37	6.8E-06
BivProm2	Weak bivalent promoter- stronger on H3K27me3	43	1.76	0.00057
TxEx3	Exon; H3K36me3 strong	4	0.30	0.0030
DNase1	DNase I only	13	2.41	0.0041
ReprPC1	Polycomb repressed; H3K27me3 strong and H3K4me1 weak	21	1.87	0.0065
BivProm1	Weak bivalent promoter - more balanced H3K4me3/H3K27me3	43	1.50	0.0092

Approximately 10% of CpG sites used to construct DNAm fitness biomarkers are conserved in other epigenetic clocks with 25% of the coefficients in the same direction. Fifty-six (out of 627) CpG sites are conserved in at least one other epigenetic clock; 7 in DNAmPhenoAge, 2 in DNAmAge, 15 in DNAmAgeHannum, 23 in DNAmAgeSkinBlood (Supplementary Table 8), and 14 in DNAmGrimAge. The most conserved CpG site was cg26842024; this is used in the male DNAmGaitspeed model and is in all clocks except Hannum. CpG sites were chosen in multiple DNAm fitness models corresponding to 46 coefficients to compare to other epigenetic clocks. In total, 11 coefficients were in the same direction as other clocks. The remaining 90% of the CpG sites used for DNAm fitness biomarkers suggest new areas of the epigenome to study that may be responsive to physical activity.

## DISCUSSION

DNAm biomarkers have been constructed for blood cell count [37], age [12, 13], smoking [15], and more, however, there were not yet DNAm biomarkers for fitness parameters. Our work introduces new DNAm biomarkers for the fitness parameters of maximum handgrip strength, gait speed, FEV1, and VO2max. These DNAm biomarkers represent new tools for researchers interested in studying the epigenetic components to physical fitness.

DNAm biomarkers have been improved by incorporating phenotypic information [14, 15], however, DNAm biomarkers had not yet incorporated physical fitness. DNAmFitAge provides researchers a novel indicator of biological age which combines physical fitness and epigenetic health. This biomarker integrates the established DNAm prediction of mortality risk (DNAmGrimAge) with the newly developed DNAm predictions of fitness. Higher values of DNAmGaitspeed, DNAmGripmax, DNAmFEV1, and DNAmVO2max, which reflect greater physical fitness, correspond to younger estimated biological ages in men and women. We demonstrate physically fit lifestyles have younger biological ages and fitter DNAm fitness biomarkers, which we observe in people of low to intermediate physical activity levels across five large-scale validation datasets and in male body builders who have intense, athletic exercise regimes. Furthermore, FitAgeAcceleration is strongly associated with a host of age-related conditions and predicts time-to-death and time-to-CHD across validation datasets. FitAgeAcceleration provides a novel measure of epigenetic age acceleration that is expected to be particularly sensitive to exercise interventions.

We acknowledge the following limitations. First, the DNAm fitness parameter biomarkers lead to only modest improvement to estimate fitness parameters after including age and sex as covariates in validation datasets. This reflects the relatively weak signal present in blood for fitness parameters. Because of the biomarkers’ limited correlation, DNAm fitness biomarkers should *not* replace true fitness parameters. Instead, the main benefit of our biomarkers is that they show blood epigenetic changes accompany physical fitness. These biomarkers advance the molecular understanding of exercise benefits, which we hypothesize to be most pronounced in athletic populations as illustrated in our analysis of body builders. The male body builders had a mean 2.7 year reduction in DNAmFitAge compared to controls, whereas the intermediate physically active people had at most a mean 0.33 year reduction in DNAmFitAge (WHI). Second, our DNAmVO2max biomarker was only validated in one dataset with VO2max; more research is needed to evaluate how our DNAmVO2max biomarker performs across a range of independent datasets.

Overall, DNAmGaitspeed, DNAmGripmax, DNAmFEV1, DNAmVO2max, and DNAmFitAge provide epigenetic components to evaluating a person’s physical fitness. Physically fit people have a younger DNAmFitAge and younger FitAgeAcceleration, and younger values are associated with more physical activity and better age-related outcomes. Our research suggests exercise and stronger fitness parameters are protective to DNAmFitAge in both sexes. We expect DNAmFitAge will be a useful biomarker for quantifying fitness benefits at an epigenetic level and can be used to evaluate exercise-based interventions.

## METHODS

### Study cohorts

We analyzed blood DNAm data from three datasets, Framingham Heart Study Offspring cohort (FHS, *n* = 1830), Baltimore Longitudinal Study on Aging (BLSA, *n* = 820), and novel data (Budapest, *n* = 307) to develop DNAm biomarkers of fitness parameters. In short, the FHS cohort is a cardiovascular study which followed adults from Massachusetts starting in 1948 [38]. The BLSA cohort began in 1958 studying healthy adults and the aging process [39]. Finally, Budapest is a smaller study (*n* = 307) measuring physical fitness and DNA methylation in middle to older aged adults, some of whom are current or former athletes. More details of the Budapest study can be found in Supplementary Note 1. Dataset harmonization was performed to join multiple datasets when variables were on different scales following previously developed methods [40]. In brief, datasets were rescaled to have the same mean and standard deviation for each fitness parameter by recentering and multiplying by the ratio of standard deviations.

We conducted validation analysis in an independent group of six additional datasets: two Lothian Birth Cohorts: LBC1921 (*n* = 692) and LBC1936 (*n* = 2797), Comprehensive Assessment of Long-term Effects of Reducing Intake of Energy (CALERIE, *n* = 578), InChianti (*n* = 924), Jackson Heart Study (JHS, *n* = 1746), and Women’s Health Initiative (WHI, *n* = 2117). Descriptive statistics of each dataset are presented in Supplementary Table 9. Full study descriptions for validation datasets have previously been published [41–46]. We evaluate our biomarkers in a novel Polish study which collects DNA methylation and dietary supplements in body builders and controls to assess performance in an athletic population. Additional details of the Polish study can be found in Supplementary Note 1.

### DNAm fitness parameter biomarker development

We developed DNAm biomarkers for four fitness parameters: gait speed, maximum handgrip strength (Gripmax), forced expiratory volume in 1 second (FEV1), and maximal oxygen uptake (VO2max). Gait speed, also known as walking speed, is measured in meters per second [47]. Maximum hand grip strength is a measurement of force taken in kg [3]. FEV1 measures lung function; it is the amount of air forced from the lungs in one second, measured in liters [8]. VO2max is a measure of cardiovascular health and aerobic endurance [4, 47]. It measures the volume of oxygen the body processes during incremental exercise in milliliters used in one minute of exercise per kilogram of body weight (mL/kg/min). VO2max has been regarded as the best indicator of an athlete’s physical capacity and is the international standard of physical capacity [47].

Each fitness DNAm biomarker was developed using LASSO penalized regression with 10-fold cross validation in which the fitness parameters were dependent variables and independent variables were DNAm levels at cytosine-phosphate-guanines (CpG) sites and chronological age. The LASSO-regression method uses an l_1_ penalty that shrinks each coefficient towards zero. LASSO is more effective than Ridge (l_2_ penalty) and elastic net (mixture of l_1_ and l_2_ penalty) when handling many irrelevant predictors and yields smaller number of predictors in the final model. Models were fit separately for men and women in the case of gait speed, gripmax, and FEV1 to select for sex specific CpG loci that reflect gender variation in fitness. The selected covariates and estimated coefficients were then used to form a prediction algorithm for each fitness parameter. We refer to the predicted fitness parameters generated by these algorithms as DNAmGaitspeed, DNAmGripmax, DNAmFEV1, and DNAmVO2max. Correlation of each DNAm biomarker with measured fitness values in the training data are displayed in Supplementary Figure 3.

When it came to building the biomarker for VO2max, stratifying by sex was not feasible due to the smaller sample size. This forced us to choose between using sex as a covariate or omitting sex and trusting LASSO to select X chromosome markers that best signify differences between males and females. We chose the latter, and it did. Finally, we present two models for DNAmGaitspeed and DNAmGripmax; one with chronological age and one without chronological age as potential covariates. Removing age as a potential variable for selection in LASSO was performed to remedy high collinearity discovered among these DNAm biomarkers when constructing DNAmFitAge (scatterplot matrix in Supplementary Figure 4).

### DNAm fitness parameter biomarker validation

We conducted two validation analyses of DNAm biomarkers of fitness parameters using up to five independent datasets. First, we correlated DNAm biomarker values with direct measurements of the fitness parameters. In cases where direct measurement of a fitness parameter was not included in a validation dataset, substitutions were selected. Briefly, gait speed was substituted with a composite leg strength measurement and a composite physical functioning score; FEV1 was substituted by forced expiratory volume (FEV) and VO2max; VO2max was substituted by FEV. Details are reported in Supplementary Note 2.

Second, we evaluated if using our DNAm biomarkers improve estimation of fitness parameters beyond variation explained through age and sex (null models) by evaluating the significance of the DNAm biomarker as a predictor. Pearson correlations of null models are presented in Supplementary Table 1. The reported *p*-values indicate the significance of the DNAm biomarker estimate as a predictor for the fitness parameters. The individual- dataset and fixed-effects meta-analysis *p*-values are calculated across validation datasets with the most relevant variables available in more than one dataset. Specifically, LBC1921 and LBC1936 were used for DNAmGaitSpeed and DNAmFEV1 meta-analysis *p*-value calculations. LBC1921, LBC1936, CALERIE, and WHI were used for DNAmGripmax. We did not calculate a meta-analysis *p*-value for DNAmVO2max because only one validation dataset had VO2max measurement.

### DNAmFitAge: biological age estimation

#### DNAmFitAge development

We constructed DNAmFitAge as an indicator of biological age following the methods proposed by Klemera and Doubal [48]. In brief, the Klemera-Doubal model framework stipulates there exists an underlying trait which is unobserved (biological age) which relates to an observable trait (chronological age) and a set of additional variables. This framework posits biological age is centered on chronological age with additional noise. Weighted least squares is used to estimate the relationship of the additional variables with biological age where the weights are formed from correlations of each variable with chronological age.

DNAmFitAge is constructed separately for males and females using four DNAm variables: three of the DNAm fitness biomarkers: DNAmGripmax, DNAmGaitSpeed, and DNAmVO2max, and DNAmGrimAge, a biomarker of mortality risk [15]. We estimate biological age using the TrueTrait function from the WGCNA R package which carries out the Klemera Doubal method described above. Variable weights indicating each variable’s importance for estimating biological age are presented in Table 2A. Pearson’s correlation among original fitness parameters, DNAm biomarkers, and DNAmFitAge in the large training dataset (FHS + BLSA) are displayed in Supplementary Figure 2. Pearson’s correlation of DNAmFitAge to chronological age in training data are presented in panels A and B of Supplementary Figure 2. Models including DNAmFEV1 as a fifth variable were explored, however no improvement in association to physical activity or age-related outcomes were observed; the parsimonious DNAmFitAge model using a subset of the DNAm fitness biomarkers was therefore chosen.

Finally, we created FitAgeAcceleration, the age-adjusted estimate of DNAmFitAge formed from taking the residuals after regressing DNAmFitAge onto chronological age. As such, FitAgeAcceleration is uncorrelated with chronological age. FitAgeAcceleration provides an estimate of epigenetic age acceleration, i.e., how much older or younger a person’s estimated biological age is from expected chronological age. A positive FitAgeAcceleration means biological age is estimated to be older than chronological age. A negative FitAgeAcceleration means biological age is estimated to be younger than chronological age, which is the preferred outcome for a person.

#### DNAmFitAge validation

DNAmFitAge validation analysis consisted of three components: correlating DNAmFitAge to chronological age, testing FitAge Acceleration association with physical activity, and testing FitAge Acceleration association to aging-related variables in the validation datasets. First, the modeling framework posits biological age is centered on chronological age, therefore validation datasets should demonstrate good correlation and general centeredness between DNAmFitAge and chronological age. Both properties would indicate DNAmFitAge can quantify age. Second, DNAmFitAge incorporates fitness, therefore FitAgeAcceleration (age adjusted DNAmFitAge) should relate to physical activity and physical functioning. These relationships would indicate DNAmFitAge relates to fitness. Third, DNAmFitAge provides insight to the aging process through a fitness paradigm, therefore FitAgeAcceleration should relate to aging-related phenotypes.

We correlate DNAmFitAge with chronological age for males and females because (1) we cannot directly measure biological age, (2) chronological age is not used when forming DNAmFitAge estimates, and (3) the modeling framework posits biological age is centered on chronological age. In addition, because DNAmFitAge is built in males and females separately, we demonstrate what happens when the model is applied to the opposite sex (i.e., male model in females or female model in males). Median absolute deviation, mean deviation, and Pearson correlation are presented in Supplementary Table 2 and correlation is displayed in Figure 2.

We tested for associations between physical activity or physical functioning in low to intermediate physically fit individuals with FitAgeAcceleration, DNAm fitness parameter biomarkers, and other DNAm biomarkers known to relate to physical health. We restricted our analysis to people of low to intermediate fitness to determine if FitAgeAcceleration is more sensitive to small improvements in fitness compared to other current DNAm biomarkers. In addition, this separation captures low to average physically active individuals in each dataset. In short, LBC1921, LBC1936, and JHS measure physical activity, and WHI and InChianti measure physical functioning. Higher values of any variable indicate more activity or better physical functioning. Other DNAm biomarkers which relate to physical health include DNAmPhenoAge [14], DNAmGrimAge, DNAmPAI-1, and DNAmGDF-15 [15]. See Supplementary Note 2 for a thorough description of physical activity variables and inclusion criteria. We use *p*-values across models to compare DNAm biomarker performance, however other methods could be used that may be more valuable, like likelihood ratio tests or AIC. We chose not to use other methods because of high collinearity among the DNAm biomarkers and the succinctness of *p*-values.

We tested DNAmFitAge associations to multiple aging-related variables in validation datasets. Specifically, we conducted regression analysis of physical activity, time-to-death, time-to-coronary-heart-disease (CHD), the count of age-related conditions (arthritis, cataract, cancer, CHD, CHF, emphysema, glaucoma, lipid condition, osteoporosis, and type 2 diabetes), age at menopause, cancer, hypertension, type-2 diabetes, and disease-free status. Time-to-event outcomes were analyzed using Cox regression to estimate hazard ratios (HR); continuous outcomes were analyzed using linear regression to estimate slopes; dichotomous outcomes were analyzed using logistic regression to estimate odds ratios (OR); and ordinal outcomes were analyzed using multinomial regression to estimate OR. Some of our cohorts (InChianti, LBC1921, and LBC1936) involved longitudinal measures. In these cases, linear regression models with person-level random intercepts were implemented in R using the lmer function to adjust for correlation within the same individual. Logistic regression models were estimated using generalized estimating equations with the R function gee. Multinomial models were implemented using R function multinom.

FitAgeAcceleration was also explored for explaining information in time-to-death and number of comorbidities beyond what is captured through other epigenetic clocks. DNAmFitAge is built using DNAmGrimAge, and DNAmGrimAge and other epigenetic clocks are known to be associated with age-related conditions. Therefore, FitAgeAcceleration (the age-adjusted measure) is compared to other epigenetic biomarkers using a Likelihood Ratio Test (LRT) in two nested models stratified by sex; one includes age and one other epigenetic clock, and the other includes age, the other epigenetic clock, and FitAge Acceleration. LRTs and corresponding *p*-values are presented for validation datasets in Supplementary Table 4 for DNAmGrimAge, DNAmPAI1, DNAmGDF15, DNAmAgeHannum, and DNAmAgeSkinBloodClock. We excluded LRTs for other health related outcomes (like disease free status) because those models are constructed from generalized estimating equations (GEE) which are not based on likelihoods, and likelihoods are necessary to compare LRTs.

### Meta-analysis

We combine results across validation studies using fixed effect models or Stouffer’s meta analysis method using the metafor R function. Fixed effect models use the inverse variance to weight estimates, and Stouffer’s method uses the square root of the sample size to weight estimates. The latter is used when harmonization across cohorts was challenging; such as with physical activity variables, the number of age-related conditions, disease free status, and age at menopause. Forest plots evaluating FitAgeAcceleration hazard ratios or coefficients in models adjusted for age and sex are displayed in Figure 3 and Supplementary Table 3. We perform a test of heterogeneity for coefficients across datasets using Cochran Q test for fixed effect models; *p*-values are displayed as Het. P.

### DNAmFitAge evaluation in body builders

We evaluated whether our DNAm fitness biomarkers and DNAmFitAge were significantly different in an independent study of male body builders and controls. There was a total of 66 male body builders and 149 male controls with similar age distributions (*p*-value > 0.05). Both groups reported the number of years they regularly trained, the average number of intensity trainings they participated in per week, and 88 total participants reported supplements or drugs they are taking. We analyzed whether the DNAm fitness biomarkers, DNAmFitAge, or FitAge Acceleration were different between male controls and body builders using a Kruskal Wallis test (Table 4).

We evaluate whether the improvement in DNAmFitAge and DNAmVO2max in male body builders can be explained by the dietary supplements taken using a linear regression model with DNAmFitAge or DNAmVO2max as the outcome with age as a covariate and indicator variables for taking the supplement and being a body builder. We adjust for age in the model because age was significantly related to taking certain supplements, therefore if age was not included, the differences observed in DNAmFitAge or DNAmVO2max may actually represent differences in chronological ages between supplement usage groups. Linear model results are presented in Supplementary Table 5. To ensure adequate power, we evaluated supplements and drugs with at least 10 people reporting use across both body builders and controls. Only six supplements met this threshold: multivitamins (*n* = 19), protein (*n* = 17), energy (*n* = 17) (creatine, pre-workout, and energy gels), magnesium (*n* = 16), vitamin D (*n* = 14), and omega-3 (*n* = 12). We also evaluated if these supplements were disproportionately taken by male body builders compared to male controls using Fisher’s Exact test (Supplementary Table 6).

### Functional CpG annotation

We provide biological insight to the 627 unique CpG loci used in constructing our DNAm fitness biomarkers by exploring genomic enrichment in the entire human genome, analyzing specific enrichment in chromatin states, and comparing CpG loci and coefficients to other epigenetic clocks. We use the GREAT enrichment analysis software tool for analyzing broad genomic enrichment [49]. GREAT analyzes the genes within and nearby the genomic region covered by the CpGs. To avoid confounding the enrichment analysis by gene size, the GREAT algorithm performs a binomial test (over genomic regions) using a whole genome background. We performed the enrichment based on default settings (Proximal: 5.0 kb upstream, 1.0 kb downstream, plus Distal: up to 1,000 kb) using the hg19 assembly. We report nominal, Bonferroni, and FDR *p*-values for gene, biological, cellular, and molecular function in Table 5A for the top results, and complete results are presented in Supplementary Table 7.

To annotate the CpGs used to construct the DNAm fitness biomarkers based on chromatin state, we assigned a state for the CpGs based on the detailed universal ChromHMM chromatin state annotation of the human genome in which chromatin structure and their associated characteristics are annotated [34]. This annotation generated 100 distinct states using 1,032 experiments into 16 major categories such as active and weak enhancers (EnhA, EnhW), bivalent states associated with promoters (BivProm), flanking promoter states (PromF), polycomb repressed states associated with H3K27me3 (ReprPC), and states associated with exons and transcription. We used one-sided hypergeometric tests to study both the enrichment (OR >1) and depletion (OR <1) patterns of CpGs across the chromatin states as detailed in [50]. Genomic CpG regions on the 450K array with chromatin state information were used as background (*n* = 483,090). The genomic regions of DNAm fitness biomarker CpG sites with chromatin state information were used as foreground (*n* = 626), which only excluded 1 CpG. This yielded one-sided hypergeometric *p*-values not confounded by the number of CpGs within a gene. We report the chromatin state, number of CpG loci enriched in each state, Odds Ratios, and hypergeometric *p*-values in Table 5B, and complete results are presented in Supplementary Table 10. Because the underlying chromatin states follow a multinomial distribution, we do not adjust our *p*-values for multiple comparisons.

Finally, we compared the CpG loci and model coefficients used in construction of the DNAm fitness biomarkers to the CpG loci used in DNAmPhenoAge, DNAmAge (Horvath 2013), DNAmAgeHannum, and DNAmAgeSkinBlood. For CpG loci conserved across other epigenetic clocks, we report the DNAm fitness biomarker coefficients, other clock coefficients, overlap with other clocks, and whether the coefficient direction is the same in Supplementary Table 8. We also compare the overlap of CpG loci used in our models and DNAmGrimAge but omit the comparison of coefficient direction to prevent disclosure of intellectual property. For a full list of coefficients and CpG loci used in DNAm fitness biomarker construction, see our GitHub repository at https://github.com/kristenmcgreevy/DNAmFitAge.

## Supplementary Materials

Supplementary Materials

Supplementary Figures

Supplementary Tables

## References

[1] Harridge SD, Lazarus NR. Physical Activity, Aging, and Physiological Function. Physiology (Bethesda). 2017; 32:152–61. 10.1152/physiol.00029.2016 28228482

[2] Agusti A, Faner R. Lung function trajectories in health and disease. Lancet Respir Med. 2019; 7:358–64. 10.1016/S2213-2600(18)30529-0 30765254

[3] Frederiksen H, Hjelmborg J, Mortensen J, McGue M, Vaupel JW, Christensen K. Age trajectories of grip strength: cross-sectional and longitudinal data among 8,342 Danes aged 46 to 102. Ann Epidemiol. 2006; 16:554–62. 10.1016/j.annepidem.2005.10.006 16406245

[4] Rapp D, Scharhag J, Wagenpfeil S, Scholl J. Reference values for peak oxygen uptake: cross-sectional analysis of cycle ergometry-based cardiopulmonary exercise tests of 10 090 adult German volunteers from the Prevention First Registry. BMJ Open. 2018; 8:e018697. 10.1136/bmjopen-2017-018697 29506981 PMC5855221

[5] van Oostrom SH, Engelfriet PM, Verschuren WMM, Schipper M, Wouters IM, Boezen M, Smit HA, Kerstjens HAM, Picavet HSJ. Aging-related trajectories of lung function in the general population-The Doetinchem Cohort Study. PLoS One. 2018; 13:e0197250. 10.1371/journal.pone.0197250 29768509 PMC5955530

[6] Jackson AS, Sui X, Hébert JR, Church TS, Blair SN. Role of lifestyle and aging on the longitudinal change in cardiorespiratory fitness. Arch Intern Med. 2009; 169:1781–7. 10.1001/archinternmed.2009.312 19858436 PMC3379873

[7] Radak Z, Torma F, Berkes I, Goto S, Mimura T, Posa A, Balogh L, Boldogh I, Suzuki K, Higuchi M, Koltai E. Exercise effects on physiological function during aging. Free Radic Biol Med. 2019; 132:33–41. 10.1016/j.freeradbiomed.2018.10.444 30389495

[8] Ching SM, Chia YC, Lentjes MAH, Luben R, Wareham N, Khaw KT. FEV1 and total Cardiovascular mortality and morbidity over an 18 years follow-up Population-Based Prospective EPIC-NORFOLK Study. BMC Public Health. 2019; 19:501. 10.1186/s12889-019-6818-x 31053065 PMC6500069

[9] Rezwan FI, Imboden M, Amaral AFS, Wielscher M, Jeong A, Triebner K, Real FG, Jarvelin MR, Jarvis D, Probst-Hensch NM, Holloway JW. Association of adult lung function with accelerated biological aging. Aging (Albany NY). 2020; 12:518–42. 10.18632/aging.102639 31926111 PMC6977706

[10] Lazarus NR, Lord JM, Harridge SDR. The relationships and interactions between age, exercise and physiological function. J Physiol. 2019; 597:1299–309. 10.1113/JP277071 30422311 PMC6395415

[11] Huggett DL, Connelly DM, Overend TJ. Maximal aerobic capacity testing of older adults: a critical review. J Gerontol A Biol Sci Med Sci. 2005; 60:57–66. 10.1093/gerona/60.1.57 15741284

[12] Horvath S. DNA methylation age of human tissues and cell types. Genome Biol. 2013; 14:R115. 10.1186/gb-2013-14-10-r115 24138928 PMC4015143

[13] Hannum G, Guinney J, Zhao L, Zhang L, Hughes G, Sadda S, Klotzle B, Bibikova M, Fan JB, Gao Y, Deconde R, Chen M, Rajapakse I, et al. Genome-wide methylation profiles reveal quantitative views of human aging rates. Mol Cell. 2013; 49:359–67. 10.1016/j.molcel.2012.10.016 23177740 PMC3780611

[14] Levine ME, Lu AT, Quach A, Chen BH, Assimes TL, Bandinelli S, Hou L, Baccarelli AA, Stewart JD, Li Y, Whitsel EA, Wilson JG, Reiner AP, et al. An epigenetic biomarker of aging for lifespan and healthspan. Aging (Albany NY). 2018; 10:573–91. 10.18632/aging.101414 29676998 PMC5940111

[15] Lu AT, Quach A, Wilson JG, Reiner AP, Aviv A, Raj K, Hou L, Baccarelli AA, Li Y, Stewart JD, Whitsel EA, Assimes TL, Ferrucci L, Horvath S. DNA methylation GrimAge strongly predicts lifespan and healthspan. Aging (Albany NY). 2019; 11:303–27. 10.18632/aging.101684 30669119 PMC6366976

[16] Marioni RE, Shah S, McRae AF, Chen BH, Colicino E, Harris SE, Gibson J, Henders AK, Redmond P, Cox SR, Pattie A, Corley J, Murphy L, et al. DNA methylation age of blood predicts all-cause mortality in later life. Genome Biol. 2015; 16:25. 10.1186/s13059-015-0584-6 25633388 PMC4350614

[17] Chen BH, Marioni RE, Colicino E, Peters MJ, Ward-Caviness CK, Tsai PC, Roetker NS, Just AC, Demerath EW, Guan W, Bressler J, Fornage M, Studenski S, et al. DNA methylation-based measures of biological age: meta-analysis predicting time to death. Aging (Albany NY). 2016; 8:1844–65. 10.18632/aging.101020 27690265 PMC5076441

[18] Belsky DW, Caspi A, Corcoran DL, Sugden K, Poulton R, Arseneault L, Baccarelli A, Chamarti K, Gao X, Hannon E, Harrington HL, Houts R, Kothari M, et al. DunedinPACE, a DNA methylation biomarker of the pace of aging. Elife. 2022; 11:e73420. 10.7554/eLife.73420 35029144 PMC8853656

[19] Horvath S, Erhart W, Brosch M, Ammerpohl O, von Schönfels W, Ahrens M, Heits N, Bell JT, Tsai PC, Spector TD, Deloukas P, Siebert R, Sipos B, et al. Obesity accelerates epigenetic aging of human liver. Proc Natl Acad Sci U S A. 2014; 111:15538–43. 10.1073/pnas.1412759111 25313081 PMC4217403

[20] Soerensen M, Li W, Debrabant B, Nygaard M, Mengel-From J, Frost M, Christensen K, Christiansen L, Tan Q. Epigenome-wide exploratory study of monozygotic twins suggests differentially methylated regions to associate with hand grip strength. Biogerontology. 2019; 20:627–47. 10.1007/s10522-019-09818-1 31254144 PMC6733812

[21] Ferrari L, Vicenzi M, Tarantini L, Barretta F, Sironi S, Baccarelli AA, Guazzi M, Bollati V. Effects of Physical Exercise on Endothelial Function and DNA Methylation. Int J Environ Res Public Health. 2019; 16:2530. 10.3390/ijerph16142530 31315170 PMC6678332

[22] Spólnicka M, Pośpiech E, Adamczyk JG, Freire-Aradas A, Pepłońska B, Zbieć-Piekarska R, Makowska Ż, Pięta A, Lareu MV, Phillips C, Płoski R, Żekanowski C, Branicki W. Modified aging of elite athletes revealed by analysis of epigenetic age markers. Aging (Albany NY). 2018; 10:241–52. 10.18632/aging.101385 29466246 PMC5842850

[23] Hughes DC, Ellefsen S, Baar K. Adaptations to Endurance and Strength Training. Cold Spring Harb Perspect Med. 2018; 8:a029769. 10.1101/cshperspect.a029769 28490537 PMC5983157

[24] Studenski S, Perera S, Patel K, Rosano C, Faulkner K, Inzitari M, Brach J, Chandler J, Cawthon P, Connor EB, Nevitt M, Visser M, Kritchevsky S, et al. Gait speed and survival in older adults. JAMA. 2011; 305:50–8. 10.1001/jama.2010.1923 21205966 PMC3080184

[25] Bohannon RW. Grip Strength: An Indispensable Biomarker For Older Adults. Clin Interv Aging. 2019; 14:1681–91. 10.2147/CIA.S194543 31631989 PMC6778477

[26] Clausen JSR, Marott JL, Holtermann A, Gyntelberg F, Jensen MT. Midlife Cardiorespiratory Fitness and the Long-Term Risk of Mortality: 46 Years of Follow-Up. J Am Coll Cardiol. 2018; 72:987–95. 10.1016/j.jacc.2018.06.045 30139444

[27] Babb TG, Long KA, Rodarte JR. The relationship between maximal expiratory flow and increases of maximal exercise capacity with exercise training. Am J Respir Crit Care Med. 1997; 156:116–21. 10.1164/ajrccm.156.1.9511021 9230734

[28] Rahmania SK, Prabowo T, Tessa P. Correlation between Forced Expiratory Volume One Second and Vital Capacity with VO2 Maximum. Althea Med J. 2016; 3:430–3. https://pdfs.semanticscholar.org/d5ae/a86cad41b87f5168886c13b93b0c9fb62065.pdf.

[29] Fatemi R, Ghanbarzadeh M. Relationship Between Airway Resistance indices and Maximal Oxygen Uptake in Young Adults. J Hum Kinet. 2009; 22:29–34. 10.2478/v10078-009-0020-7

[30] Ekelund U, Tarp J, Steene-Johannessen J, Hansen BH, Jefferis B, Fagerland MW, Whincup P, Diaz KM, Hooker SP, Chernofsky A, Larson MG, Spartano N, Vasan RS, et al. Dose-response associations between accelerometry measured physical activity and sedentary time and all cause mortality: systematic review and harmonised meta-analysis. BMJ. 2019; 366:l4570. 10.1136/bmj.l4570 31434697 PMC6699591

[31] Vigneron N, Peaper DR, Leonhardt RM, Cresswell P. Functional significance of tapasin membrane association and disulfide linkage to ERp57 in MHC class I presentation. Eur J Immunol. 2009; 39:2371–6. 10.1002/eji.200939536 19701894 PMC3517023

[32] Silverman MN, Deuster PA. Biological mechanisms underlying the role of physical fitness in health and resilience. Interface Focus. 2014; 4:20140040. 10.1098/rsfs.2014.0040 25285199 PMC4142018

[33] Munters LA, Loell I, Ossipova E, Raouf J, Dastmalchi M, Lindroos E, Chen YW, Esbjörnsson M, Korotkova M, Alexanderson H, Nagaraju K, Crofford LJ, Jakobsson PJ, Lundberg IE. Endurance Exercise Improves Molecular Pathways of Aerobic Metabolism in Patients With Myositis. Arthritis Rheumatol. 2016; 68:1738–50. 10.1002/art.39624 26867141

[34] Vu H, Ernst J. Universal annotation of the human genome through integration of over a thousand epigenomic datasets. Genome Biol. 2022; 23:9. 10.1186/s13059-021-02572-z 34991667 PMC8734071

[35] Margueron R, Reinberg D. The Polycomb complex PRC2 and its mark in life. Nature. 2011; 469:343–9. 10.1038/nature09784 21248841 PMC3760771

[36] Lu AT, Fei Z, Haghani A, Robeck TR, Zoller JA, Li CZ, Zhang J, Ablaeva J, Adams DM, Almunia J, Ardehali R, Arneson A, Baker CS, et al. Universal DNA methylation age across mammalian tissues. bioRxiv. 2021; 1–28. 10.1101/2021.01.18.426733 PMC10501909 37563227

[37] Houseman EA, Accomando WP, Koestler DC, Christensen BC, Marsit CJ, Nelson HH, Wiencke JK, Kelsey KT. DNA methylation arrays as surrogate measures of cell mixture distribution. BMC Bioinformatics. 2012; 13:86. 10.1186/1471-2105-13-86 22568884 PMC3532182

[38] Dawber TR, Meadors GF, Moore FE Jr. Epidemiological approaches to heart disease: the Framingham Study. Am J Public Health Nations Health. 1951; 41:279–81. 10.2105/ajph.41.3.279 14819398 PMC1525365

[39] Ferrucci L. The Baltimore Longitudinal Study of Aging (BLSA): a 50-year-long journey and plans for the future. J Gerontol A Biol Sci Med Sci. 2008; 63:1416–9. 10.1093/gerona/63.12.1416 19126858 PMC5004590

[40] Key TJ, Appleby PN, Allen NE, Reeves GK. Pooling biomarker data from different studies of disease risk, with a focus on endogenous hormones. Cancer Epidemiol Biomarkers Prev. 2010; 19:960–5. 10.1158/1055-9965.EPI-10-0061 20233851 PMC2875156

[41] Taylor AM, Pattie A, Deary IJ. Cohort Profile Update: The Lothian Birth Cohorts of 1921 and 1936. Int J Epidemiol. 2018; 47:1042–1042r. 10.1093/ije/dyy022 29546429 PMC6124629

[42] Stenholm S, Koster A, Valkeinen H, Patel KV, Bandinelli S, Guralnik JM, Ferrucci L. Association of Physical Activity History With Physical Function and Mortality in Old Age. J Gerontol A Biol Sci Med Sci. 2016; 71:496–501. 10.1093/gerona/glv111 26290538 PMC4834834

[43] Rickman AD, Williamson DA, Martin CK, Gilhooly CH, Stein RI, Bales CW, Roberts S, Das SK. The CALERIE Study: design and methods of an innovative 25% caloric restriction intervention. Contemp Clin Trials. 2011; 32:874–81. 10.1016/j.cct.2011.07.002 21767664 PMC3185196

[44] Waziry R, Corcoran DL, Huffman KM, Kobor MS, Kothari M, Kraus VB, Kraus WE, Lin DTS, Pieper CF, Ramaker ME, Bhapkar M, Das SK, Ferrucci L, et al. Effect of Long-Term Caloric Restriction on DNA Methylation Measures of Biological Aging in Healthy Adults: CALERIE™ Trial Analysis. medRxiv. 2021; 1–27. 10.1101/2021.09.21.21263912

[45] Taylor HA Jr, Wilson JG, Jones DW, Sarpong DF, Srinivasan A, Garrison RJ, Nelson C, Wyatt SB. Toward resolution of cardiovascular health disparities in African Americans: design and methods of the Jackson Heart Study. Ethn Dis. 2005; 15:S6-4-17. 16320381

[46] Manson JE, Chlebowski RT, Stefanick ML, Aragaki AK, Rossouw JE, Prentice RL, Anderson G, Howard BV, Thomson CA, LaCroix AZ, Wactawski-Wende J, Jackson RD, Limacher M, et al. Menopausal hormone therapy and health outcomes during the intervention and extended poststopping phases of the Women's Health Initiative randomized trials. JAMA. 2013; 310:1353–68. 10.1001/jama.2013.278040 24084921 PMC3963523

[47] Rankovic G, Mutavdzic V, Toskic D, Preljevic A, Kocic M, Nedin Rankovic G, Damjanovic N. Aerobic capacity as an indicator in different kinds of sports. Bosn J Basic Med Sci. 2010; 10:44–8. 10.17305/bjbms.2010.2734 20192930 PMC5596610

[48] Klemera P, Doubal S. A new approach to the concept and computation of biological age. Mech Ageing Dev. 2006; 127:240–8. 10.1016/j.mad.2005.10.004 16318865

[49] McLean CY, Bristor D, Hiller M, Clarke SL, Schaar BT, Lowe CB, Wenger AM, Bejerano G. GREAT improves functional interpretation of cis-regulatory regions. Nat Biotechnol. 2010; 28:495–501. 10.1038/nbt.1630 20436461 PMC4840234

[50] Lu AT, Hannon E, Levine ME, Crimmins EM, Lunnon K, Mill J, Geschwind DH, Horvath S. Genetic architecture of epigenetic and neuronal ageing rates in human brain regions. Nat Commun. 2017; 8:15353. 10.1038/ncomms15353 28516910 PMC5454371

