# Supplementary material for: DNAmFitAge: biological age indicator incorporating physical fitness
Source: Aging (Albany NY). 2023 Feb 22;15(10):3904–38. doi: 10.18632/aging.204538 (PMC10258016; doi:10.18632/aging.204538)
Supplement: Supplementary Figures [file aging-15-204538-s002.pdf]

SUPPLEMENTARY FIGURES

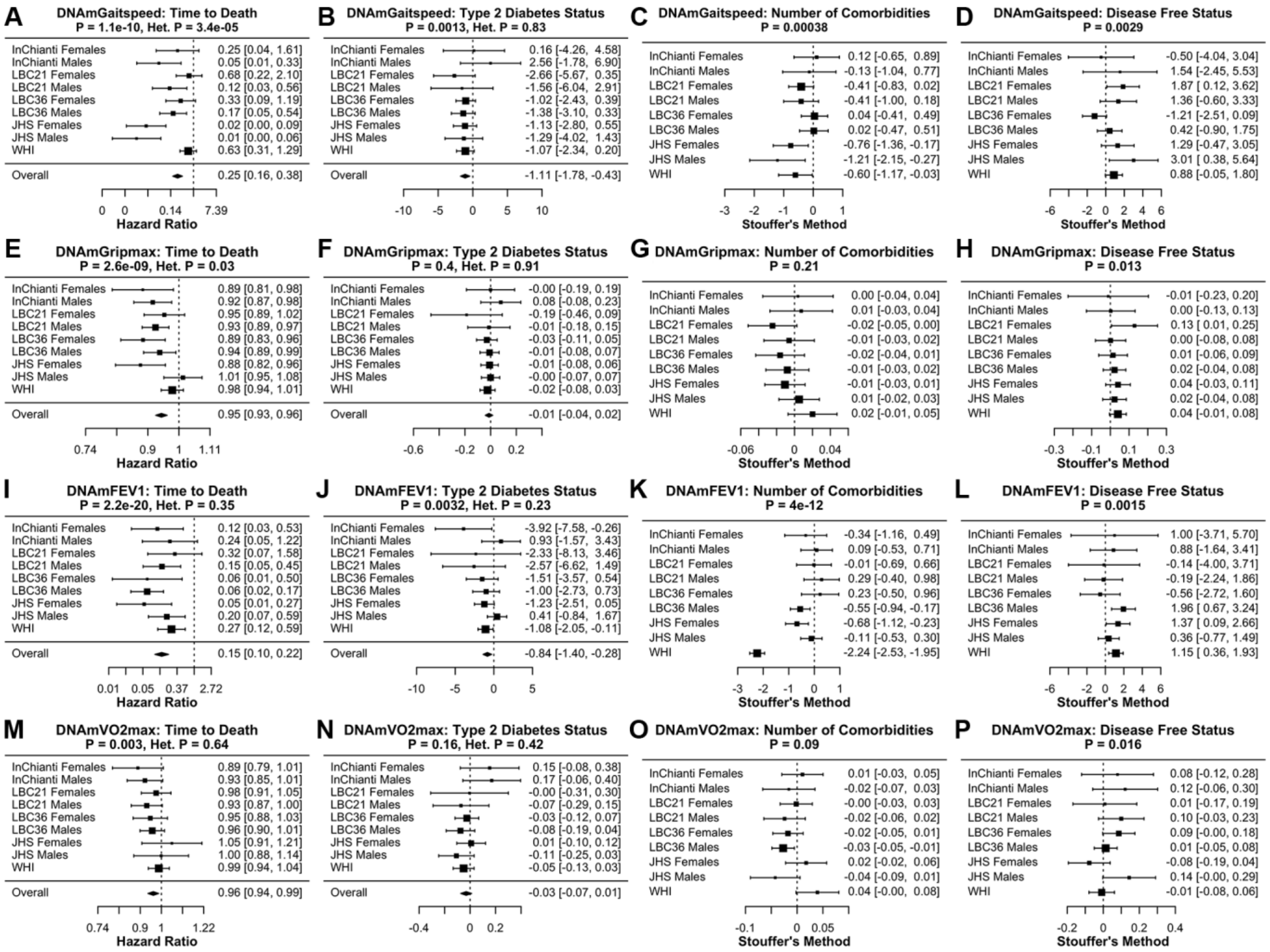

Supplementary Figure 1. Meta-analysis forest plots for DNAm fitness parameter biomarkers with age-related conditions. Each row reports a meta analysis forest plot for combining hazard ratios or regression coefficients across dataset cohorts for one DNAm biomarker estimate. (**A**–**D**) DNAmGaitSpeed without age, (**E**–**H**) DNAmGripmax without age, (**I**–**L**) DNAmFEV1, and (**M**–**P**) DNAmVO2max. Time-to-death, type 2 diabetes, comorbidity count, and disease-free status are presented. Meta-analysis *p*-values are displayed in the header of each panel, and test of heterogeneity Cochran *Q* test *p*-value (Het. P) are displayed for fixed effect models. Fixed effects models were used for time-to-death and type 2 diabetes whereas Stouffer's method was used for comorbidity count and disease-free status. All DNAm fitness biomarkers are predictive of mortality, and DNAmGaitspeed and DNAmFEV1 are predictive of number of comorbidities.

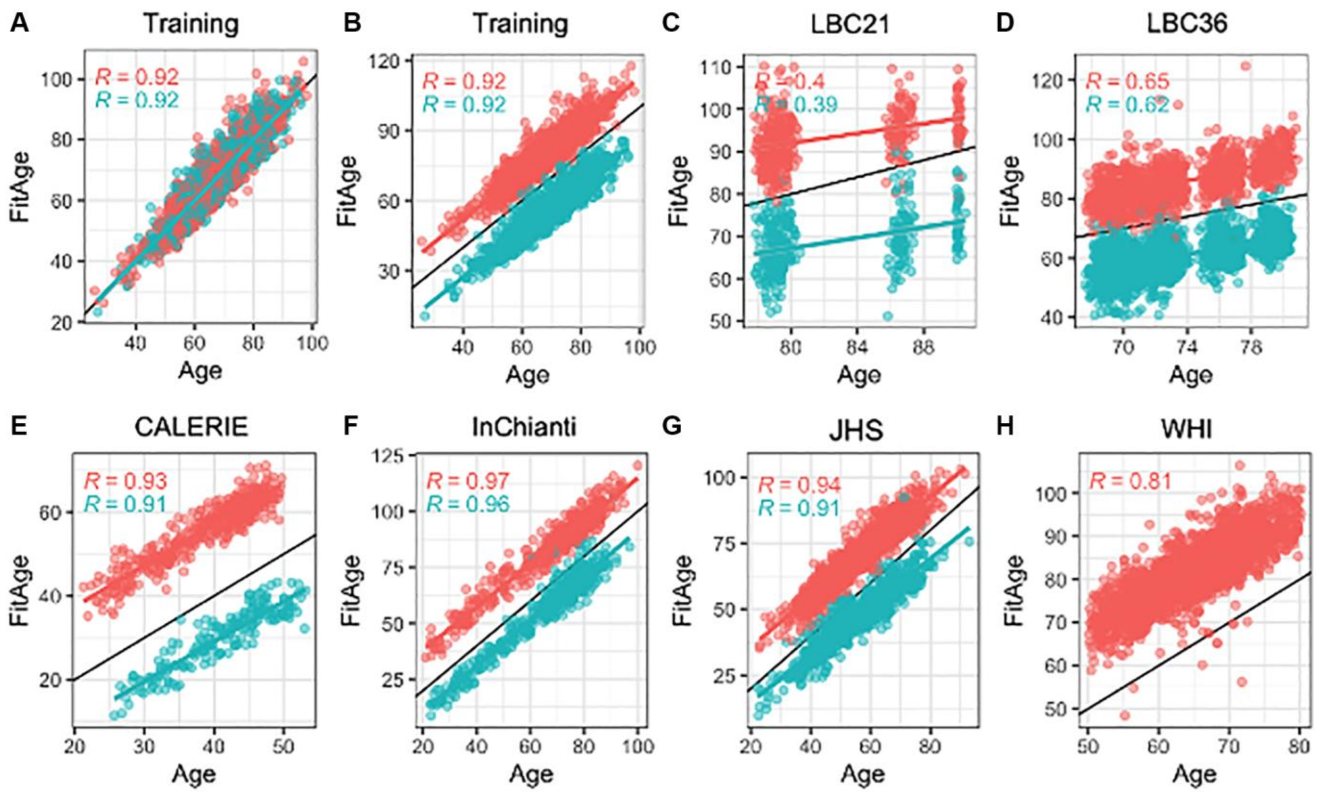

Supplementary Figure 2. Scatterplots of DNAmFitAge versus age in training and test datasets separated by sex. Pink indicates females, and blue indicates males. Each panel corresponds to the performance of DNAmFitAge across datasets displayed with Pearson correlation to chronological age and corresponding *p*-values. (**A**) DNAmFitAge performance in training dataset built in each sex separately. (**B**–**H**) DNAmFitAge models applied to the opposite sex it was built in (ie DNAmFitAge built for females tested in males and DNAmFitAge built for males tested in females). Females are estimated to be older than they are, and males are estimated to be younger than they are in training and test datasets.

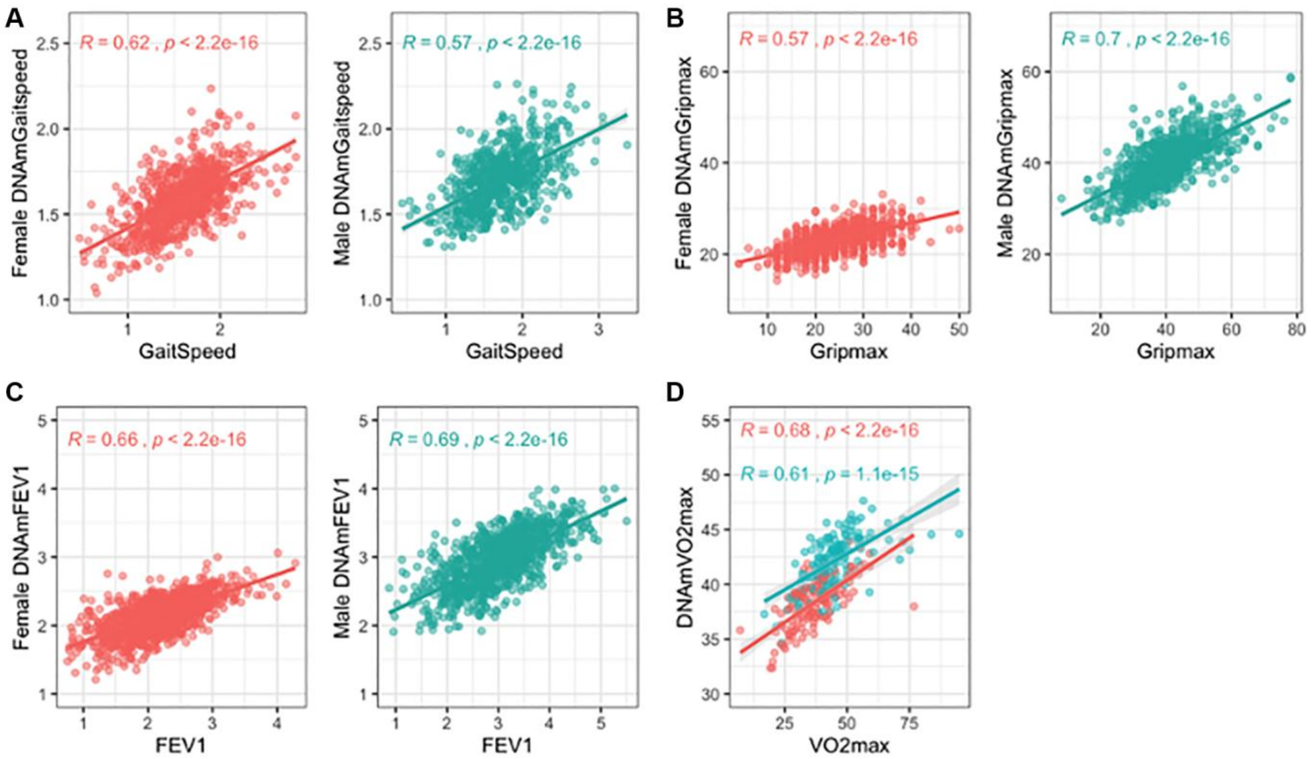

Supplementary Figure 3. Scatterplots of DNAm fitness biomarker models versus true fitness values in training data. Pink indicates females, and blue indicates males. Each panel corresponds to the training performance of one DNAm biomarker model built with chronological age displayed with Pearson correlation and *p*-values. (**A**) DNAmGaitspeed, (**B**) DNAmGripmax, (**C**) DNAmFEV1, and (**D**) DNAmVO2max. (**A**–**C**) (DNAmGaitspeed, DNAmGrip, and DNAmFEV1) were built in each sex separately while (**D**) (DNAmVO2max) was built in both sexes jointly.

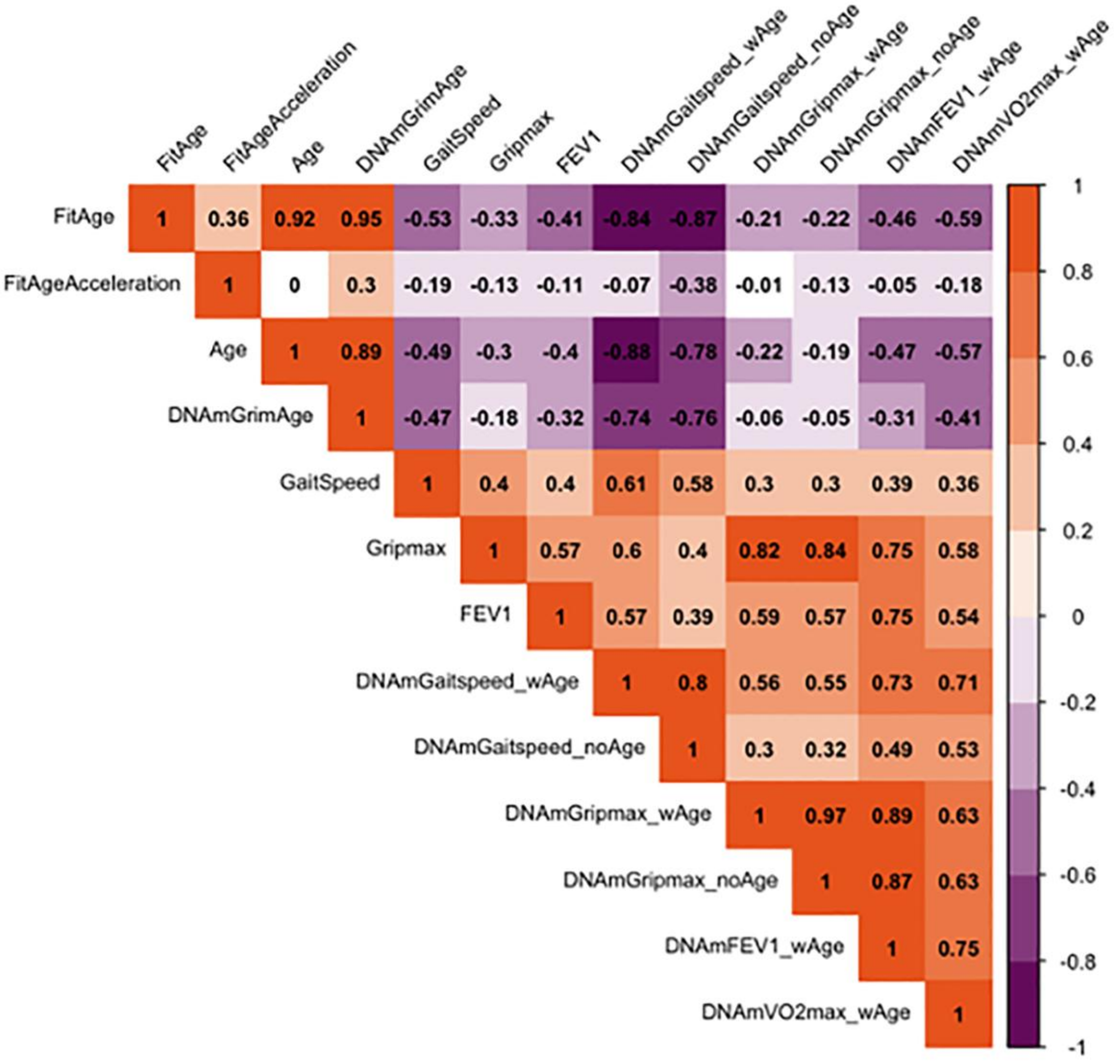

Supplementary Figure 4. Correlation matrix in largest training data (FHS and BLSA) among fitness parameters, DNAm fitness parameter biomarkers, and DNAmFitAge. Pearson r correlation between two variables are displayed. Correlation among VO2max is not displayed because this variable was not present in the large training data. Variables labeled "_wAge" indicate models built with chronological age as a predictor, and variables labeled "_noAge" indicate models built using only CpG loci (ie without chronological age as a predictor).
