# Supplementary material for: DNAmFitAge: biological age indicator incorporating physical fitness
Source: Aging (Albany NY). 2023 Feb 22;15(10):3904–38. doi: 10.18632/aging.204538 (PMC10258016; doi:10.18632/aging.204538)
Supplement: Supplementary Tables [file aging-15-204538-s003.pdf]

SUPPLEMENTARY TABLES

Supplementary Table 1. Validation dataset null model pearson correlation and DNAm biomarker significance.

DNAm Biomarker	Sex	Age in Model	LBC1921		LBC1936		CALERIE		InChianti		WHI		Meta Analysis *p*-value
			Null R	DNAm *p*-value	Null R	DNAm *p*-value	Null R	DNAm *p*-value	Null R	DNAm *p*-value	Null R	DNAm *p*-value	
Gaitspeed	Females	Y	0.412	0.437	0.382	0.118	0.100*	0.519	0.478^{+}	0.011	0.107^{+}	1.7E-05	0.082
		N		0.069		0.230		0.736		0.427		0.0002	0.051
	Males	Y	0.418	0.092	0.413	0.118	0.264*	0.519	0.334^{+}	0.011			0.0048
		N		0.011		0.230		0.736		0.427			0.0037
Gripmax	Females	Y	0.422	0.556	0.114	7.7E-05	0.170	0.258			0.128^{+}	0.012	0.056
		N		0.0014		0.028		0.312				0.430	0.056
	Males	Y	0.363	0.026	0.203	0.034	0.115	0.095					0.702
		N		0.0037		0.005		0.939					0.0015
FEV1	Females	Y	0.163$^{\wedge}$	0.0017	0.214$^{\wedge}$	0.0061	0.193	4.3E-05					1.99E-04
	Males	Y	0.196$^{\wedge}$	0.0001	0.206$^{\wedge}$	9.2E-09	0.483	0.655					2.01E-09
VO2max	Overall	Y	0.623$^{\wedge}$	0.0065	0.606$^{\wedge}$	0.809	0.706	0.00013					-

*Composite Leg Strength, $^{+}$Physical Functioning, $^{\wedge}$FEV

Supplementary Table 2. DNAmFitAge to chronological age performance in validation datasets.

		Females	Males	Male model in females	Female model in males
Training Data	Median Absolute Deviation	2.7	3.0	11.9	13.5
	Mean Deviation	0.0	0.0	−12.2	13.1
	R	0.923	0.925	0.925	0.922
LBC1921	Median Absolute Deviation	3.7	4.8	11.0	14.5
	Mean Deviation	0.8	1.1	−11.1	13.8
	R	0.409	0.386	0.404	0.391
LBC1936	Median Absolute Deviation	3.2	3.4	11.6	13.3
	Mean Deviation	0.0	0.2	−11.9	12.9
	R	0.635	0.635	0.647	0.624
CALERIE	Median Absolute Deviation	4.9	2.3	17.1	11.0
	Mean Deviation	−5.0	−2.0	−17.1	11.0
	R	0.926	0.915	0.928	0.912
InChianti	Median Absolute Deviation	3.9	3.9	16.0	9.6
	Mean Deviation	−3.8	−4.3	−16.1	9.1
	R	0.969	0.964	0.969	0.963
JHS	Median Absolute Deviation	2.9	3.4	13.6	9.2
	Mean Deviation	−1.6	−2.8	−13.9	8.6
	R	0.937	0.917	0.940	0.914
WHI	Median Absolute Deviation	3.8		16.8	
	Mean Deviation	−3.4		−16.8	
	R	0.808		0.812	

Supplementary Table 3. FitAgeAcceleration association to phenotypic outcomes.

Meta analysis *p*-values		Time to death*	Time to coronary heart disease*	Type 2 diabetes	Number of comorbidities	Disease free status	Total cholesterol	Age at menopause	Any cancer	hypertension
		***p* = 7.2 E-51**	***p* = 2.6 E-8**	***p* = 2.7 E-9**	***p* = 9.0 E-9**	***p* = 1.1 E-7**	***p* = 0.00048**	***p* = 6.6 E-9**	***p* = 0.157**	***p* = 8.7 E-5**
LBC1921 Females	Coefficients	1.03		0.042	0.007	0.009	-0.039			
	p-values	0.013		0.230	0.171	0.922	0.0017			
	No. of events	375		16	96	266	-			
LBC1921 Males	Coefficients	1.06		0.033	0.007	−0.027	-0.027			
	p-values	1.62E-06		0.392	0.273	0.161	0.029			
	No. of events	277		21	102	157	-			
LBC1936 Females	Coefficients	1.08		0.046	0.008	−0.006	-0.010	−0.081		
	p-values	3.76E-08		0.0015	0.153	0.672	0.112	0.043		
	No. of events	334		109	1171	179	-	-		
LBC1936 Males	Coefficients	1.09		0.048	0.017	−0.038	-0.011			
	p-values	9.24E-12		0.0032	0.0031	0.0048	0.045			
	No. of events	499		183	1219	211	-			
InChianti Females	Coefficients	1.06		0.018	−0.002	−0.006	-0.046	−2.96	−0.059	0.0037
	p-values	0.011		0.635	0.842	0.868	0.223	0.00034	0.176	0.881
	No. of events	167		33	235	104	-	-	37	140
InChianti Males	Coefficients	1.07		−0.070	2.11E-05	−0.045	−0.004		0.045	0.026
	p-values	1.01E-06		0.135	0.998	0.215	0.905		0.292	0.219
	No. of events	162		33	221	87	-		31	143
WHI	Coefficients	1.05	1.04	0.050	0.021	−0.031	−0.008	−0.060	0.025	0.024
	p-values	8.06E-09	1.20E-05	0.00052	0.0011	0.0041	0.570	3.82E-05	0.063	0.014
	No. of events	771	2117	392	1539	793	-	-	388	918
JHS Females	Coefficients	1.15	1.13	0.054	0.029	−0.062	−0.116	0.747**		0.057
	p-values	1.96E-15	0.00025	0.0016	4.12E-07	0.00024	0.696	0.152		0.00071
	No. of events	156	1001	267	721	352	-	-		664
JHS Males	Coefficients	1.06	1.06	0.012	0.012	−0.034	−1.148			0.021
	p-values	9.25E-07	0.041	0.495	0.066	0.082	0.0006			0.232
	No. of events	125	579	135	406	226	-			363

*Hazard Ratios; **Not age at menopause, menopause status; number of events for comorbidities is people with at least 1 comorbidity.

Supplementary Table 4. Comparing DNAmFitAge importance with other DNAm biomarkers for time-to-death and number of comorbidities after controlling for age and sex.

		LBC1921		LBC1936		InChianti		WHI		JHS	
**Time-to-Death Model Comparison**		**LRT**	**LRT *p*-value**	**LRT**	**LRT *p*-value**	**LRT**	**LRT *p*-value**	**LRT**	**LRT *p*-value**	**LR T**	**LRT *p*-value**
DNAmGrimAge + DNAmFitAge to DNAmGrimAge	Females	0.5	0.479	2.9	0.091	7.2	0.007	1.1	0.286	4.6	0.032
	Males	3.7	0.054	2.6	0.110	7.7	0.005			0.2	0.628
DNAmPhenoAge + DNAmFitAge to DNAmPhenoAge	Females	9.1	0.003	36.0	1.98E-09	1.2	0.269	17.0	3.70E-05	30.4	3.53E-08
	Males	11.3	7.64E-04	91.0	< 1.0E-16	26.4	2.76E-07			4.3	0.039
DNAmPAI1 + DNAmFitAge to DNAmPAI1	Females	9.8	0.002	51.1	8.67E-13	7.3	0.007	17.0	3.76E-05	38.5	5.36E-10
	Males	30.4	3.49E-08	83.6	< 1.0E-16	22.9	1.67E-06			7.1	0.008
DNAmGDF15 + DNAmFitAge to DNAmGDF15	Females	5.2	0.023	44.8	2.16E-11	6.1	0.014	23.1	1.54E-06	46.6	8.88E-12
	Males	25.0	5.78E-07	70.7	< 1.0E-16	14.1	1.73E-04			4.4	3.66E-02
DNAmAgeHannum + DNAmFitAge to DNAmAgeHannum	Females	13.2	2.79E-04	60.8	6.11E-15	2.0	0.157	31.5	1.95E-08	41.6	1.11E-10
	Males	15.0	1.09E-04	104.0	< 1.0E-16	22.0	2.70E-06			7.0	0.008
DNAmAgeSkinBlood Clock + DNAmFitAge to DNAmAgeSkinBlood Clock	Females	16.4	5.05E-05	92.3	< 1.0E-16	3.6	0.058	34.7	3.79E-09	57.2	3.89E-14
	Males	22.4	2.17E-06	133.8	< 1.0E-16	21.8	3.01E-06			10.9	9.46E-04
Number of Comorbidities Model Comparison											
DNAmGrimAge + DNAmFitAge to DNAmGrimAge	Females	2.1	0.148	1.2	0.269	0.4	0.513	3.4	0.065	0.0	0.910
	Males	2.5	0.117	3.1	0.080	0.05	0.828			1.2	0.267
DNAmPhenoAge + DNAmFitAge to DNAmPhenoAge	Females	0.05	0.828	2.6	0.110	3.4	0.067	2.9	0.091	8.6	0.003
	Males	5.2	0.023	38.7	4.98E-10	0.01	0.927			0.7	0.412
DNAmPAI1 + DNAmFitAge to DNAmPAI1	Females	0.7	0.401	1.3	0.255	0.9	0.344	1.4	0.230	2.7	0.101
	Males	1.4	0.233	26.5	2.70E-07	0.1	0.800			0.1	0.817
DNAmGDF15 + DNAmFitAge to DNAmGDF15	Females	0.5	0.476	5.3	0.021	0.01	0.944	6.7	0.010	22.1	2.61E-06
	Males	2.6	0.105	31.6	1.86E-08	0.6	0.453			4.2	0.041
DNAmAgeHannum + DNAmFitAge to DNAmAgeHannum	Females	0.03	0.871	2.4	0.123	0.7	0.411	6.5	0.011	13.1	2.88E-04
	Males	2.6	0.108	39.2	3.78E-10	0.2	0.624			1.1	0.305
DNAmAgeSkinBlood Clock + DNAmFitAge to DNAmAgeSkinBlood Clock	Females	0.3	0.596	5.6	0.018	0.2	0.682	8.3	0.004	16.5	4.83E-05
	Males	1.3	0.256	55.0	1.21E-13	0.2	0.676			2.9	0.089

Supplementary Table 5. Linear models evaluating dietary supplement usage to DNAmFitAge and DNAmVO2max in males after adjusting for age.

Supplement in model		Outcome: DNAmFitAge		Outcome: DNAmVO2max	
		**Supplement**	**BodyBuilder**	**Supplement**	**BodyBuilder**
Multivitamins	coefficient	–0.32	–0.62	0.68	0.07
	p-value	0.690	0.208	0.041	0.746
Proteins	coefficient	–0.05	–0.65	0.45	0.10
	p-value	0.961	0.184	0.241	0.607
Energy	coefficient	0.16	–0.66	0.24	0.13
	p-value	0.852	0.175	0.518	0.513
Magnesium	coefficient	–1.03	–0.60	-0.12	0.15
	p-value	0.213	0.219	0.727	0.472
Vitamin D	coefficient	–0.56	–0.62	-0.32	0.16
	p-value	0.570	0.207	0.439	0.431
Omega-3	coefficient	–1.23	–0.46	0.33	0.08
	p-value	0.157	0.366	0.355	0.687

Supplementary Table 6. Dietary supplement use by male athlete status.

		**Control**	**Body builder**	**Fisher's exact *p*-value**
Multivitamins	No	141	55	0.016
	Yes	8	11	
Proteins	No	140	58	0.169
	Yes	9	8	
Energy	No	145	53	6.81E-05
	Yes	4	13	
Magnesium	No	140	59	0.265
	Yes	9	7	
Vitamin D	No	143	58	0.036
	Yes	6	8	
Omega-3	No	144	59	0.050

Supplementary Table 7. Complete GREAT analysis CpG annotation.

Genes	Observed Regions	Fold Enrichment	Binomial *p*-value	Bonferroni *p*-value	FDR Q-value
ZNRD1	4	77.9	2.8E-07	0.0051	0.0051
HLA-G	4	55.0	1.1E-06	0.020	0.010
KCNS1	3	93.3	5.4E-06	0.100	0.033
HOXA2	2	518.0	7.4E-06	0.138	0.034
TAP2	2	421.0	1.1E-05	0.208	0.042
Cellular					
MHC protein complex	9	25.1	1.9E-10	3.1E-07	3.1E-07
integral component of endoplasmic reticulum membrane	21	3.7	4.5E-07	7.5E-04	3.7E-04
intrinsic component of endoplasmic reticulum membrane	21	3.6	6.4E-07	0.0011	3.5E-04
MHC class II protein complex	5	26.9	1.6E-06	0.0026	6.5E-04
integral component of lumenal side of endoplasmic reticulum membrane	7	12.7	1.8E-06	0.0030	6.0E-04
MHC class I protein complex	4	23.1	3.2E-05	0.054	0.0089
TAP complex	2	195.9	5.2E-05	0.086	0.012
DNA-directed RNA polymerase I complex	5	12.2	6.7E-05	0.112	0.014
MHC class I peptide loading complex	2	169.4	6.9E-05	0.115	0.013
A band	10	4.2	1.7E-04	0.286	0.029
lysosomal membrane	30	2.0	2.9E-04	0.479	0.044
Molecular					
peptide antigen binding	6	13.3	7.7E-06	0.032	0.032
tapasin binding	2	421.0	1.1E-05	0.047	0.023
MHC class II receptor activity	4	28.5	1.4E-05	0.060	0.020
antigen binding	11	4.8	2.8E-05	0.116	0.029
peptide antigen-transporting ATPase activity	2	249.6	3.2E-05	0.133	0.027
DNA-directed 5′–3′ RNA polymerase activity	8	6.7	3.5E-05	0.148	0.025
5′–3′ RNA polymerase activity	8	6.5	4.4E-05	0.185	0.026

Supplementary Table 8. DNAm fitness CpG loci overlap with other epigenetic clocks.

	CpG	DNAm Fitness Coefficient	Clocks Present in	Same Coefficient Direction	PhenoAge Coefficient (*n* = 7)	DNAmAge Coefficient (*n* = 2)	Hannum Coefficient (*n* = 15)	Skin and Blood Coefficient (*n* = 23)	DNAm Fitness Model
1	cg26842024	−0.16	3	0	0.06	0.36		0.48	DNAmGaitspeed Males no Age
2	cg00748589	−0.37	2	0			8.21	0.17	DNAmGaitspeed Males no Age
3	cg02867102	0.32	2	0			−12.50	-0.12	DNAmGaitspeed Males no Age
4	cg03607117	−0.63	2	0			10.70	0.17	DNAmGaitspeed Females no Age
		−6.47		0			10.70	0.17	DNAmGripmax Females no Age
5	cg04424621	0.01	2	1	-14.45			0.04	DNAmGaitspeed Males no Age
6	cg04875128	−4.72	2	1			−4.37	0.0004	DNAmGripmax Males no Age
7	cg06639320	−8.60	2	0			8.95	0.02	DNAmGripmax Males no Age
8	cg07082267	0.22	2	1			2.87	-0.47	DNAmGaitspeed Males no Age
9	cg10917602	0.02	2	0	-2.82			-0.02	DNAmGaitspeed Females w/ Age
10	cg16419235	−0.23	2	1			−1.60	0.11	DNAmGaitspeed Females no Age
		−3.81		1			−1.60	0.11	DNAmGripmax Females no Age
11	cg16867657	−0.21	2	0			10.80	0.90	DNAmGaitspeed Males no Age
		−1.26		0			10.80	0.90	DNAmGaitspeed Females no Age
		−8.32		0			10.80	0.90	DNAmGripmax Males no Age
		−12.72		0			10.80	0.90	DNAmGripmax Females no Age
12	cg20822990	1.53	2	0			−15.70	−0.01	DNAmGripmax Males no Age
13	cg22454769	−0.17	2	0			4.85	0.05	DNAmGaitspeed Males no Age
14	cg23500537	−0.22	2	0			5.67	0.23	DNAmGripmax Males no Age
15	cg25410668	−1.90	2	0			3.87	0.28	DNAmGripmax Males no Age
16	cg26581729	4.08	2	1	-4.00			0.02	DNAmGripmax Males no Age
17	cg00481951	−0.52	1	1			−2.72		DNAmGaitspeed Males no Age
18	cg05228408	0.05	1	0	-4.49				DNAmGaitspeed Females w/ Age
19	cg07502389	−0.36	1	0				0.32	DNAmGaitspeed Females no Age
20	cg08622677	−4.43	1	0				0.23	DNAmGripmax Females no Age
21	cg12753631	0.11	1	0				−0.34	DNAmGaitspeed Males no Age
22	cg12864235	0.06	1	1	0.66				DNAmFEV1 Females w/ Age
23	cg13649056	−4.04	1	0				0.04	DNAmGripmax Females no Age
		−0.43		0				0.04	DNAmGaitspeed Females no Age
24	cg17110586	−0.32	1	1				−0.07	DNAmGaitspeed Females no Age
		−7.53		1				−0.07	DNAmGripmax Females no Age
25	cg18691434	−12.20	1	0	4.54				DNAmGripmax Males no Age
		−0.13		0	4.54				DNAmGaitspeed Males no Age
		−3.40		0	4.54				DNAmGripmax Males w/ Age
26	cg18933331	0.06	1	0				−0.23	DNAmGaitspeed Males no Age
27	cg19702785	−0.23	1	0				0.13	DNAmGaitspeed Males no Age
28	cg22285878	−4.14	1	1			−20.70		DNAmGripmax Females no Age
29	cg25101936	0.004	1	0		−0.06			DNAmGaitspeed Females w/ Age
30	ch.13.39564907R	0.30	1	0			−20.60		DNAmGaitspeed Males no Age

Supplementary Table 9. Descriptive statistics for each dataset.

	BLSA	Budapest	LBC1921	LBC1936	CALERIE	InChianti	JHS	WHI	Polish
Total Observations	820	307	692	2797	578	924	1746	2117	215
Age mean (sd)	69.2 (13.6)	60.3 (11.7)	82.3 (4.3)	73.6 (3.7)	39.4 (7.2)	67.0 (16.6)	56.2 (12.3)	65.4 (7.1)	35.5 (8.8)
< 40	24 (3%)	8 (3%)	0 (0%)	0 (0%)	265 (46%)	100 (11%)	173 (10%)	0 (0%)	144 (67%)
40–59	178 (22%)	133 (43%)	0 (0%)	0 (0%)	313 (54%)	128 (14%)	856 (49%)	525 (25%)	71 (33%)
60–79	400 (49%)	151 (50%)	410 (59%)	2719 (97%)	0 (0%)	502 (54%)	691 (40%)	1589 (75%)	0 (0%)
80+	218 (27%)	15 (5%)	282 (41%)	78 (3%)	0 (0%)	194 (21%)	26 (2%)	3 (0%)	0 (0%)
Sex									
Males	417 (51%)	148 (48%)	291 (42%)	1141 (52%)	178 (31%)	426 (46%)	649 (37%)	0 (0%)	215 (100%)
Females	403 (50%)	159 (52%)	401 (58%)	1356 (49%)	400 (69%)	498 (54%)	1097 (63%)	2117 (100%)	0 (0%)
Race									
White	572 (70%)	297 (97%)	692 (100%)	2797 (100%)	442 (77%)	924 (100%)	0 (0%)	1007 (48%)	215 (100%)
Black	216 (26%)	0 (0%)	0 (0%)	0 (0%)	71 (12%)	0 (0%)	1746 (100%)	677 (32%)	0 (0%)
Asian	24 (3%)	10 (3%)	0 (0%)	0 (0%)	unknown	0 (0%)	0 (0%)	unknown	0 (0%)
Other	8 (1%)	0 (0%)	0 (0%)	0 (0%)	65 (11%)	0 (0%)	0 (0%)	433 (21%)	0 (0%)

Supplementary Table 10. Complete chromatin state analysis.

	State	Number of CpG loci	Odds Ratio	Hypergeometric *p*-value		State	Number of CpG loci	Odds Ratio	Hypergeometric *p*-value
1	PromF4	25	0.45	6.5E-06	51	Acet1	5	1.43	0.277
2	TSS1	15	0.37	6.8E-06	52	EnhA1	8	1.30	0.280
3	BivProm2	43	1.76	0.00057	53	EnhA16	4	1.49	0.286
4	TxEx3	4	0.30	0.0030	54	HET9	0	0.00	0.287
5	DNase1	13	2.41	0.0041	55	EnhA12	2	0.56	0.312
6	ReprPC1	21	1.87	0.0065	56	GapArtf2	1	2.64	0.316
7	BivProm1	43	1.50	0.0092	57	znf2	2	1.74	0.320
8	ReprPC5	18	1.87	0.011	58	HET3	1	2.47	0.334
9	EnhA3	9	2.53	0.011	59	Tx2	1	0.44	0.336
10	TxWk1	0	0.00	0.014	60	EnhWk2	8	1.22	0.338
11	PromF6	11	2.13	0.018	61	HET8	1	0.44	0.338
12	EnhA8	11	2.09	0.020	62	TxEx1	2	0.59	0.342
13	ReprPC7	21	1.66	0.021	63	Acet4	5	1.29	0.346
14	PromF2	17	1.70	0.028	64	EnhA14	3	0.67	0.350
15	BivProm4	12	1.82	0.039	65	Quies4	3	1.43	0.352
16	EnhA18	11	1.87	0.039	66	Tx1	3	1.40	0.364
17	Acet2	0	0.00	0.042	67	Acet3	3	0.69	0.367
18	TxEnh5	6	0.49	0.044	68	PromF5	25	0.91	0.368
19	TxEnh2	0	0.00	0.047	69	ReprPC3	3	0.69	0.374
20	TxEnh8	8	2.02	0.049	70	PromF3	23	0.91	0.380
21	BivProm3	20	1.51	0.053	71	TxWk2	10	0.86	0.382
22	TxEx2	4	0.44	0.054	72	EnhA9	5	1.23	0.386
23	EnhWk8	2	0.32	0.055	73	ReprPC9	3	1.30	0.408

24	EnhWk6	5	2.42	0.060	74	EnhWk1	1	0.50	0.408
25	EnhWk4	0	0.00	0.083	75	EnhA11	1	0.50	0.408
26	Tx7	0	0.00	0.086	76	ReprPC4	4	0.78	0.418
27	Quies3	4	0.49	0.089	77	Acet6	2	0.66	0.420
28	ReprPC6	5	0.53	0.096	78	Acet7	4	0.78	0.420
29	Quies2	0	0.00	0.102	79	TSS2	23	0.93	0.422
30	TxEnh1	0	0.00	0.106	80	Tx4	1	0.52	0.425
31	EnhA4	6	1.84	0.114	81	TxEnh6	2	0.67	0.427
32	EnhA2	2	0.39	0.119	82	EnhA17	6	1.11	0.456
33	EnhA15	5	1.86	0.136	83	EnhA10	3	0.78	0.464
34	TxEx4	6	1.74	0.136	84	PromF7	2	0.71	0.465
35	TxEnh7	8	1.59	0.139	85	TxEnh4	8	1.06	0.482
36	Tx3	6	1.72	0.142	86	HET7	4	0.85	0.489
37	EnhA6	1	0.30	0.150	87	EnhA19	6	1.03	0.529
38	HET5	0	0.00	0.185	88	Tx5	5	1.03	0.534
39	HET6	7	1.50	0.191	89	Acet8	3	1.07	0.534
40	Tx8	1	0.34	0.204	90	HET2	10	0.96	0.538
41	Quies1	0	0.00	0.208	91	GapArtf1	1	0.68	0.566
42	HET1	5	1.59	0.210	92	ReprPC2	5	0.96	0.579
43	EnhWk3	2	0.48	0.215	93	TxEnh3	3	0.93	0.595
44	Tx6	0	0.00	0.222	94	EnhA13	1	0.73	0.601
45	EnhA7	8	1.38	0.233	95	Quies5	1	1.03	0.623
46	Acet5	1	0.36	0.235	96	EnhA20	0	0.00	0.630
47	PromF1	12	1.26	0.251	97	znf1	1	0.79	0.638
48	EnhA5	4	1.56	0.257	98	ReprPC8	1	0.85	0.672
49	EnhWk5	3	1.68	0.266	99	EnhWk7	1	0.94	0.714
50	HET4	1	3.16	0.272	100	GapArtf3	0	0.00	0.720
